# A multi-component reaction for covalent immobilization of lipases on amine-functionalized magnetic nanoparticles: production of biodiesel from waste cooking oil

**DOI:** 10.1186/s40643-022-00552-0

**Published:** 2022-05-30

**Authors:** Yalda Amini, Mansour Shahedi, Zohreh Habibi, Maryam Yousefi, Maryam Ashjari, Mehdi Mohammadi

**Affiliations:** 1 Department of Organic Chemistry and Oil, Faculty of Chemistry, Shahid Beheshti University, Tehran, Iran; 2 Nanobiotechnology Research Center, Avicenna Research Institute, ACECR, Tehran, Iran; 3 Bioprocess Engineering Department, Institute of Industrial and Environmental Biotechnology, National Institute of Genetic Engineering and Biotechnology (NIGEB), Tehran, Iran

**Keywords:** Biodiesel, Lipase, Multi-component reaction, Covalent immobilization, Magnetic nanoparticles

## Abstract

A new approach was used for the immobilization of *Thermomyces lanuginosus* lipase (TLL), *Candida antarctica* lipase B (CALB), and *Rhizomucor miehei* lipase (RML) on amine-functionalized magnetic nanoparticles (Fe_3_O_4_@SiO_2_-NH_2_) via a multi-component reaction route (using cyclohexyl isocyanide). The used method offered a single-step and very fast process for covalent attachment of the lipases under extremely mild reaction conditions (25 °C, water, and pH 7.0). Rapid and simple immobilization of 20 mg of RML, TLL, and CALB on 1 g of the support produced 100%, 98.5%, and 99.2% immobilization yields, respectively, after 2 h of incubation. The immobilized derivatives were then used for biodiesel production from waste cooking oil. Response surface methodology (RSM) in combination with central composite rotatable design (CCRD) was employed to evaluate and optimize the biodiesel production. The effect of some parameters such as catalyst amount, reaction temperature, methanol concentration, water content for TLL or water-adsorbent for RML and CALB, and ratio of *t*-butanol (wt%) were investigated on the fatty acid methyl esters (FAME) yield.

**Graphical Abstract:**

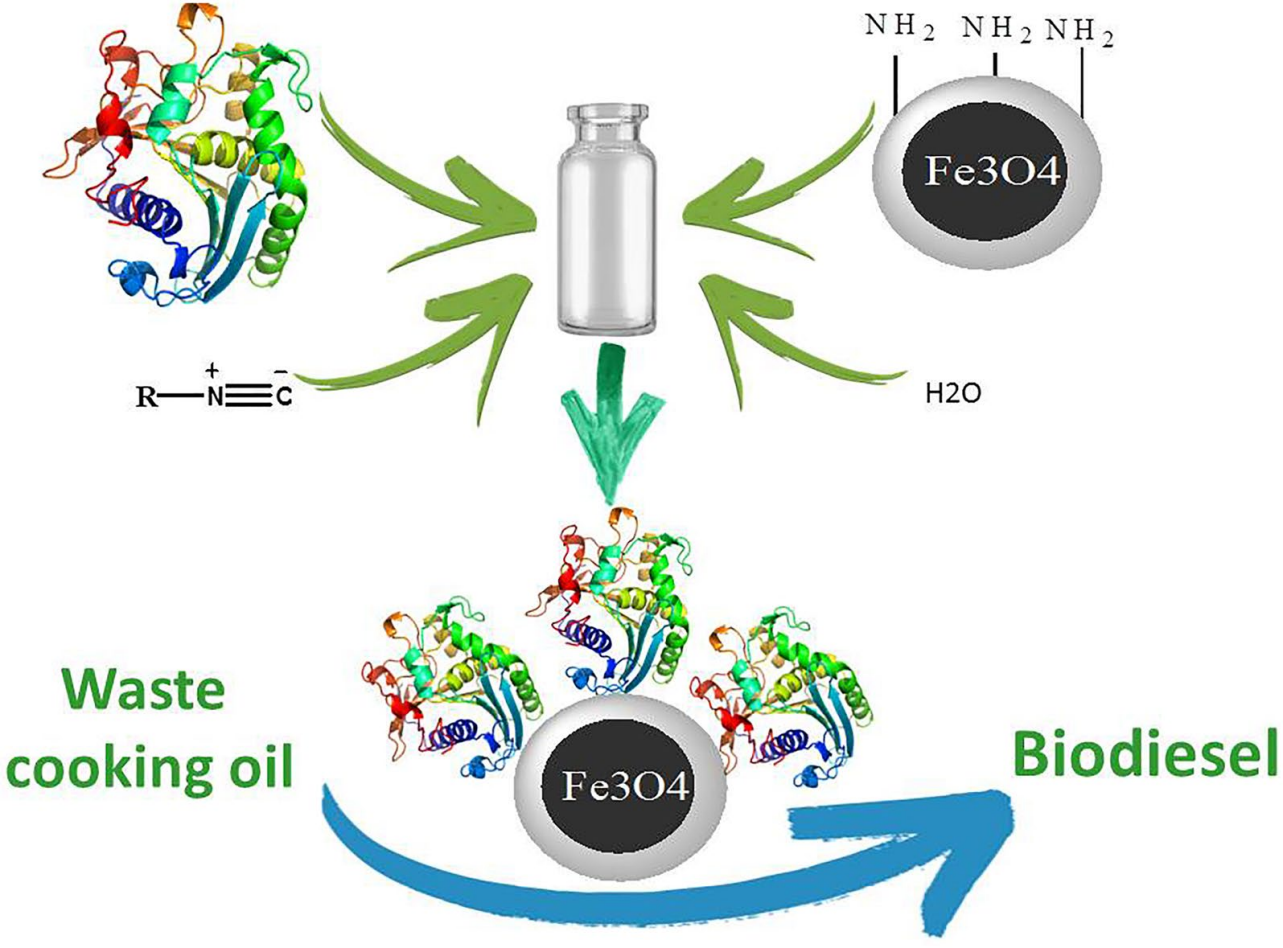

**Supplementary Information:**

The online version contains supplementary material available at 10.1186/s40643-022-00552-0.

## Introduction

The exhaustion of fossil fuels is synchronized with an accelerative rise in oil prices which augments environmental concerns. These challenges led the researches to explore renewable resources and environment-friendly fuels (Luna et al. 2014). In the next few decades, it would be required to apply biomass for the applications that are up to now produced from fossil resources, such as coal, natural gas, and oil (Franssen et al. 2013). From an economic point of view, the replacement of fossil-based chemicals by biomass sources would be attractive since various components of biomass contain molecular functionalities that are now presented in the oil-derived base chemicals with high costs (Shimada et al. 2002). Biodiesel is mono-alkyl esters of long-chain fatty acids (Mehde et al. 2018), derived from renewable feedstocks such as vegetable oils and animal fats. The key sources of vegetable oil for biodiesel production are soybean oil, sunflower oil, palm oil and rapeseed oil (Yücel 2012). Biodiesel has better lubricating properties and a much higher cetane value than petroleum diesel (Marchetti et al. 2007). Biodiesel is produced in a transesterification process in which glycerides convert into mono-alkyl esters. Transesterification processes could be classified based on chemical (alkaline-catalyzed and acid-catalyzed) or enzymatic methods (Handayani et al. 2016; Budhwani et al. 2019). The alkaline-catalyzed transesterification as the most used method for industrial production of biodiesel has several drawbacks such as the difficult recovery of glycerol and catalyst, the necessity of high energy, reaction saponification, and wastewater treatment issues (Hama et al. 2018). Enzymatic routes by using lipases (triacylglycerol acylhydrolase, EC 3.1.1.3), on the other hand, are normally conducted under mild conditions in terms of temperature, pH, and pressure (Gross et al. 2010). Lipase-catalyzed transesterification can be an excellent substitute to produce biodiesel due to the fact that it has been reported to give high yields, have simple purification of products, and need less energy consumption with a decreased amount of wastewater (Tacias-Pascacio et al. 2019; Ali et al. 2017; Ali et al. 2017). There have been very limited commercial lipases applicable in industry in their free forms, owing to the high cost and the fact that their separation, recovery and reuse are challenging. One of the most cost-effective methods adopted to eliminate these barriers is immobilization of lipases on a solid support and making them more stable heterogeneous biocatalyst (Yushkova et al. 2019; Wu et al. 2017). Immobilization of enzymes on solid carriers can be achieved by encapsulation, covalent bond and adsorption (Barbosa et al. 2015; Garmroodi et al. 2016). The strong binding between the enzyme and its carrier matrix in covalent attachment method prevents the problem of leaching and enhance the stability and reusability of the enzyme derivatives (Babaki et al. 2015). Recently, our group has introduced the use of multi-component reactions for covalent immobilization of lipases on various supports (Mohammadi et al. 2016; Habibi et al. 2013; Ashjari et al. 2020b, 2020a; Ahrari et al. 2022). Enzyme immobilization via multi-component reactions offers a very fast process in an extremely mild condition with great flexibility of accepting variety of functional groups (COOH, NH_2_, and epoxide). It means that by utilizing this procedure, enzymes can be covalently attached on a large number of functionalized matrixes. Among them, the amine-functionalized supports are known as the most widely used carriers for immobilization of enzymes (Mohammadi et al. 2018; Sigurdardóttir et al. 2018; Vashist et al. 2014). Covalent binding of enzymes on these supports is usually performed via (1) modification of amine groups by glutaraldehyde as a bifunctional linker which produces a reversible iminium bonds and requires subsequent reduction by a reducing agent like NaBH4. This 2-step procedure is time-consuming and the chemicals used may have deleterious effect on enzyme activity (Zucca and Sanjust 2014) by using diimide-activated amidation of carboxylic acids on the surface of enzyme (Jiang et al. 2004). This process is also complicated in practice and requires multiple and time-consuming steps. Besides, in this approach carboxylic groups are activated by carbodiimides which are quite costly and toxic reagents (Zucca and Sanjust 2014). These reagents also cause undesirable side reactions of intermolecular conjugation of proteins (Gao and Kyratzis 2008).

Nanoparticles have been extensively considered for enzymes immobilization (Cipolatti et al. 2014; Ghosh et al. 2012). There are some characteristics related to the nanoparticle-based biocatalysts: (i) they can be easily synthesized in high solid content without surfactants and toxic reagents; (ii) homogeneous and well defined core–shell nanoparticles with a thick enzyme shell can be obtained and (iii) designed according to the research necessity (Ansari and Husain 2012). Since the 1970s, magnetic nanoparticles have progressively been used in the field of bioscience and medicine (Shinkai 2002; Cao et al. 2017). Surface modification is a useful approach to improve thermal stability of nude magnetic nanoparticles (MNPs) and provide available reaction sites for further functionalization. Number of modifications protocols have been reported for functionalization of magnetic nanoparticles (MNPs) surface, for example by cross-linking with glutaraldehyde, coating with polymers (Jiang et al. 2007) or coupling with compounds like as agarose (He et al. 2014).

In this research superparamagnetic Fe_3_O_4_ nanoparticles coated with layer of silica were prepared and subsequently functionalized by amine functional groups. The prepared support was then used for the immobilization of *Thermomyces lanuginosus* (TLL), *Rhizomucor miehei* (RML) as 1,3-specific lipases, and *Candida antarctica* lipase B (CALB) as a non-specific lipase via a multi-component reaction. FT-IR, SEM, and XRD were used to characterize the support before and after immobilization. Ultimately, application of the immobilized lipases in the synthesis of biodiesel was evaluated. Response surface methodology (RSM) that is a powerful and efficient mathematical approach was applied for the process optimization (Yücel 2012; Ghadge and Raheman 2006; Jang et al. 2012). A 5-level 5-factor central composite design (CCD) was employed to design the experiments. The effect of several reaction parameters including water content (for TLL), water-adsorbent (for RML and CALB), reaction time, *t*-butanol concentration, temperature, and the amount of biocatalyst were optimized.

## Materials and methods

### Materials

Lipases from *Thermomyces lanuginosus* (TLL), *Rhizomucor miehei* (RML), *Candida antarctica* lipase B (CALB), FeCl_2_.4H_2_O, FeCl_3_.6H_2_O, *p*-nitrophenyl butyrate (*p*-NPB), and tetraethyl orthosilicate (TEOS) were purchased from Sigma-Aldrich. Waste cooking oil was also obtained from a local restaurant and the fatty acids composition of waste cooking oil was determined to be 40.6% oleic acid, 17.58% linoleic acid, 32.24% palmitic acid, and 5.23% stearic acid. The molecular weight of the waste cooking oil which was determined from the saponification value of 196.2 mg KOH/g and acid value of 76 mg KOH/g was calculated to be 856.3 g/mol. Water content in the oil measured by Karl Fischer (Verhoef et al. 1978) titration method was determined to be 0.01% (w/w). Solvents used including *n*-hexane, methanol, ethanol, 1-propanol, toluene, *t*-butanol and blue silica gel were prepared from Merck chemicals. Triethylamine (Et_3_N) was from Titrachem and 3-aminopropyltrimethoxysilane (3-APTMS) 97% was purchased from Alfa Aesar. All the other chemicals were accessed commercially.

### Methods

#### Preparation of the magnetic Fe_3_O_4_ nanoparticle (MNPs)

Magnetic Fe_3_O_4_ nanoparticles were prepared by the conventional co-precipitation method (Tang et al. 2011; Hu et al. 2009). First, 1.25 g of FeCl_2_·4H_2_O and 3.4 g of FeCl_3_·6H_2_O were dissolved in 100 ml deionized water under nitrogen atmosphere and the reaction temperature was increased to 65 °C. Afterward 9 ml of ammonia solution (32%) was added by dropping funnel under vigorous stirring at room temperature for 1 h. The black precipitates were collected by an external magnetic field and washed with absolute ethyl alcohol and deionized water several times to reach the pH value of 7.0. Then, the magnetic nanoparticles were dried in a vacuum oven at 80 °C and used directly for being coated with silica.

#### Preparation of silica-coated magnetic nanoparticles

Fe_3_O_4_@SiO_2_ core–shell particles were prepared by the modified Stöber sol–gel process (Takeuchi 2017). 1.5 g of Fe_3_O_4_ was mixed with 60 ml of ethanol and 10 ml of HPLC water. This suspension was dispersed under ultra-sonication for 20 min, then 9 ml of aqueous ammonia 32% and 4.31 ml of tetraethyl orthosilicate (TEOS) were added to the suspension under nitrogen atmosphere. The mixture was stirred for 5 h at room temperature. The magnetic Fe_3_O_4_@SiO_2_ nanoparticles were separated by an external magnetic field washed with ethanol and distilled water and dried in a vacuum oven at 80 °C.

#### Amine-functionalization of silica-coated magnetic nanoparticles

The dry magnetic Fe_3_O_4_@SiO_2_ nanoparticles were functionalized with 3-aminopropyltrimethoxysilane (3-APTMS). Silica magnetic nanoparticles (1 g) were added to a two-necked flask containing 40 ml of dry toluene and 75 µl of triethylamine. Then, 1.2 ml of (3-aminopropyl) trimethoxysilane 97% (3-APTMS) was added to the mixture. The mixture was refluxed under nitrogen atmosphere and vigorously stirred at 120 °C for 3 h. The amine-functionalized magnetic nanoparticles (MNPs-NH_2_) were washed first with ethanol then with deionized water and the suspension was separated by an external magnet. The functionalized MNPs were then dried at room temperature.

#### Immobilization of lipase

To perform immobilization, 20 mg of amine-functionalized nanoparticles was added in vials containing water (pH 7.0) and dispersed under bath ultra-sonication for 20 min. Then, certain amount of enzyme (20–100 mg) was added to each sample. Immobilization of the enzymes was then started by adding different amount of cyclohexyl isocyanide under stirring at room temperature. The yield immobilization was determined by measuring the initial and final concentration of each enzyme in supernatant by using the Bradford’s method (Emami Bistgani et al. 2017). Finally, the immobilized lipase was removed by an external magnet and washed by deionize water. Control experiments were conducted in the absence of cyclohexyl isocyanide.

#### Enzyme activity assay

The activities of the soluble and immobilized enzymes were analyzed spectrophotometrically by measuring the absorbance produced by the release of *p*-nitrophenol in the hydrolysis of *p*-NPB in 25 mM sodium phosphate buffer at pH 8.0 and 25 °C at 410 nm (*ε* = 18,400 M^−1^ cm^−1^; Verhoef et al. 1978). Briefly, 0.05–0.2 ml of the lipase suspensions or solutions (without further dilution) was added to 2.5 ml of substrate solution (0.8 mM) under magnetic stirring. Spontaneous hydrolysis of *p*-NPB was measured using 2.5 ml of substrate solution (0.8 mM) in the absence of enzyme as control. Enzymatic activity is given as 1 μmol of *p*-nitrophenol released per minute per mg of the enzyme (IU) under the conditions described above.

#### Characterization of the support and the immobilized biocatalysts

The structure of Fe_3_O_4_@SiO_2_, MNPs-NH_2_, and MNPs-lipase was characterized by using infrared spectroscopy (IR), X-ray diffraction (XRD), and scanning electron microscopy (SEM). Fourier-transform infrared (FT-IR) spectra were performed on a Bruker FT-IR from 4000 to 400 cm^−1^ using the KBr pellet technique. The X-ray diffraction (XRD) patterns of samples were obtained on a STOE-STADV Powder Diffraction System. The scanned area was 1° to 80° (2*θ* value) operated at 40 kV and 40 mA using 1.54060 Cu radiation and the size and morphology of samples were determined using transmission electron microscopy (SEM) analysis with KYKY-EM3200. The images were digitized under the following files: voltage 25.0 kV; probe size 3.0 nm and magnification 20,000×–80000×.

#### Thermal stability of the free and immobilized lipases

To evaluate the thermal stability of the free and immobilized enzymes, 5 mg of the immobilized derivatives (60 mg lipase/g) were added to 500 μl phosphate buffer (25 mM, pH 7.0) and incubated at different temperatures range between 45, 50, 55, 60, 65 and 70 °C for 2 h. The activity was determined using the *p*-NPB assay.

#### Co-solvent stability of the free and immobilized lipases

The soluble lipases and 5 mg of the immobilized derivatives of RML, TLL and CALB (60 mg lipase/g) were added to 500 μl of sodium phosphate solution (25 mM, pH 7.0) including different concentrations (10, 20 and 50% v/v) of the organic solvents (methanol, ethanol, propanol). Samples were taken after 24 h and their residual activity was evaluated by the *p*-NPB assay.

#### Determination of the optimum pH activity

In order to determine the optimum pH activity of lipases, the soluble lipase and 5 mg of the immobilized derivatives (60 mg/g) were added in a total volume of 2 ml solution of 25 mM sodium phosphate buffer with various pH from 5.0 to 10.5 at 25 °C. The residual activity of each sample was analyzed by the *p*-NPB assay.

#### Leaching experiment

To assess the amount of desorbed enzymes from the surface of the support, 5 mg of the immobilized derivatives (60 mg/g) was incubated in a solution containing NaCl (1 M). The concentration of protein content in the supernatant was evaluated by using both the Bradford’s method and enzyme activity assay.

#### Biodiesel synthesis

In a typical experiment, the reaction was carried out in a 5-ml screw-capped vial including 130 mg of waste cooking oil and anhydrous methanol at various oil-to-methanol molar ratios (1:3, 1:4, 1:5, 1:6, and 1:7). The mixtures were incubated with the immobilized lipases at different temperatures (35, 40, 45, 50 and 55 °C). Methanolysis reactions were performed with different amounts of *t*-butanol, water-adsorbent (for RML and CALB) and water (for TLL). To analyze the impact of the solvent on methanolysis, the amount of *t*-butanol was varied (5, 20, 35, 50 and 65 wt. %) by the substrate weight. Reactions were performed for 72 h under constant agitation of 500 rpm. Aliquots of the reaction medium was taken at different times, mixed with methyl laurate (as an internal standard) and analyzed by GC.

#### GC analysis

The FAME yield in the reaction mixture was analyzed using gas chromatography (Thermo-Quest-Finnigon) equipped with a flame-ionization detector (FID) connected to Zebron capillary column (30 m × 0.25 mm i.d.; Phenomenex, USA.) Nitrogen was used as the carrier gas at a constant flow of 0.8 ml/min. The sample was weighed and mixed with 1000 μl of 0.8 mg/ml methyl laurate in *n*-hexane as an internal standard. Then, 0.5 μl of the diluted sample was injected into the GC. The column temperature was held at 150 °C for 1 min, raised to 210 °C at 25 °C/min, and then increased to 240 °C at 10 °C/min and kept for 8 min. The injector and detector temperatures were set at 150 and 300 °C, respectively. Equation (1) was used to calculate biodiesel yield:

1 $$C=\frac{\sum A-{A}_{\mathrm{IS}}}{{A}_{\mathrm{IS}}}\times \frac{{C}_{\mathrm{IS}}\times {V}_{\mathrm{IS}}}{m}\times 100\%.$$

In this equation, (∑A) is the total sub-peak area, (${A}_{\mathrm{IS}}$) is the internal standard sub-peak, (${C}_{\mathrm{IS}}$) the internal standard concentration in (mg/ml), (${V}_{\mathrm{IS}}$) volume of the internal standard (ml), and (m) sample mass (mg).

#### Experimental design

Optimization of the transesterification process of waste cooking oil was investigated using a 5-level, 5-factor Response Surface Central Composite Design (RSCCD) of Design Expert Software version 7.0.0 (State Ease Inc. Minneapolis, USA). The study required 45 experiments, consisting of 32 factorial points, 10 axial points, and 3 repetitive tests in central points. In addition, there were five independent identified variables, contained reaction temperature (35–55 °C), enzyme amounts (5–25 w/w %), as well as *t*-butanol concentration (5, 20, 35, 50, and 65 wt %), water for TLL (0–40%) and water absorbance for RML and CALB (20–60%). The stepwise methanol feeding was applied (oil: methanol ratio 1:3, 1:4, 1:5, 1:6 and 1:7). The level of each independent variable was considered based on previous researches (Ashjari et al. 2020b; Yang et al. 2005). In addition, the independent variables were encoded into low (−1) and high (+ 1) levels. In this paper, the value of α, the distance from the center, is fixed at 2 which makes the design rotatble.

#### Statistical analysis

The experimental data obtained from central composite design (CCD) were analyzed by response surface methodology. A quadratic equation (Eq. 2), which containing all the terms of interaction, was used to calculate the predicted values:

2 $$Y={\beta }_{0}+\sum_{i=1}^{5}{\beta }_{i}{X}_{i}+\sum_{i=1}^{5}{\beta }_{ii}{X}_{i}^{2}+{\sum }_{i=1}^{3}\sum_{j=i+1}^{5}{\beta }_{ij}{X}_{i}{X}_{j}+{\sum }_{i=1}^{5}{\upbeta }_{\mathrm{iii}}{X}_{i}^{3},$$

where (Y) is the yield of biodiesel from waste cooking oil, β_0_ the offset term, (βi) represents linear effect, (βii) represents squared effect, (βij) represents interaction effect, (Xi) represents the i-independent variable, and (Xj) represents the j-independent variable. The data were analyzed using the Design Expert program and the coefficients were calculated by F-test. All statistical steps including analysis of variance (ANOVA), regression analysis and plotting of contour plot were used to establish the optimum conditions to obtain high efficiency of methyl ester.

## Results and discussion

### Preparation, modification and characterization of functionalized MNPs

**Scheme 1.**
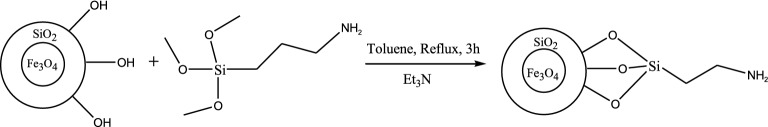
Functionalization of MNPs

Recently, MNPs have attracted remarkable attention owing to their particular properties, such as their large surface area and the presence of plenty of hydroxyl groups on their surface which enables their easy modification. The Fe_3_O_4_ magnetic nanoparticles were prepared by co-precipitation method via iron (II) and iron (III) ions. Surface modification of the activated MNPs with a layer of silica was performed by using TEOS for improving stability of MNPs and providing more available reaction sites for functionalization. Finally, the reaction of Fe_3_O_4_@SiO_2_ with (3-aminopropyl) trimethoxysilane led to amine-functionalized magnetic Fe_3_O_4_ (MNPs-NH_2_; Scheme 1). Characterization of the MNPs in various stages was performed via XRD, IR, and SEM.

**Fig. 1:**
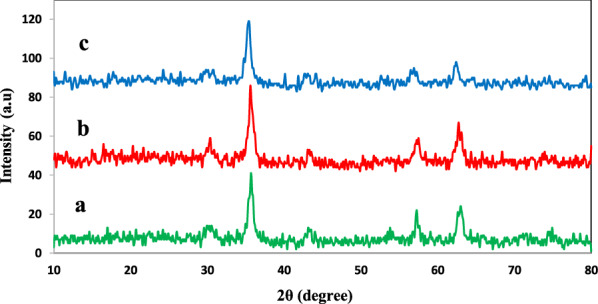
XRD patterns of **a** silica core–shell magnetic nanoparticles (Fe_3_O_4_@SiO_2_); **b** amine-functionalized magnetic nanoparticles (MNPs-NH_2_); and **c** MNPs-TLL

The X-ray diffraction patterns of Fe_3_O_4_@SiO_2_ nanoparticles (a), MNPs-NH_2_ (b) and one of the immobilized preparations (MNPs-TLL; c) were analyzed by XRD spectroscopy (Fig. 1). XRD-diagram of the bare MNPs showed spinel ferrites pattern. All the strong peaks observed at 2θ = 30.2°, 36.4°, 43.7°, 53.5°, 56.3° and 62.3° are indexed to the highly crystalline cubic spinel structure of Fe_3_O_4_ nanoparticles (Yang et al. 2005). The same sets of characteristic peaks were also observed for Fe_3_O_4_@SiO_2_, MNPs-NH_2_ and MNPs-TLL indicating the stability of the crystalline phase of Fe_3_O_4_ nanoparticles during silica coating, amine functionalization and enzyme immobilization (Ghasemzadeh et al. 2015).

**Fig. 2:**
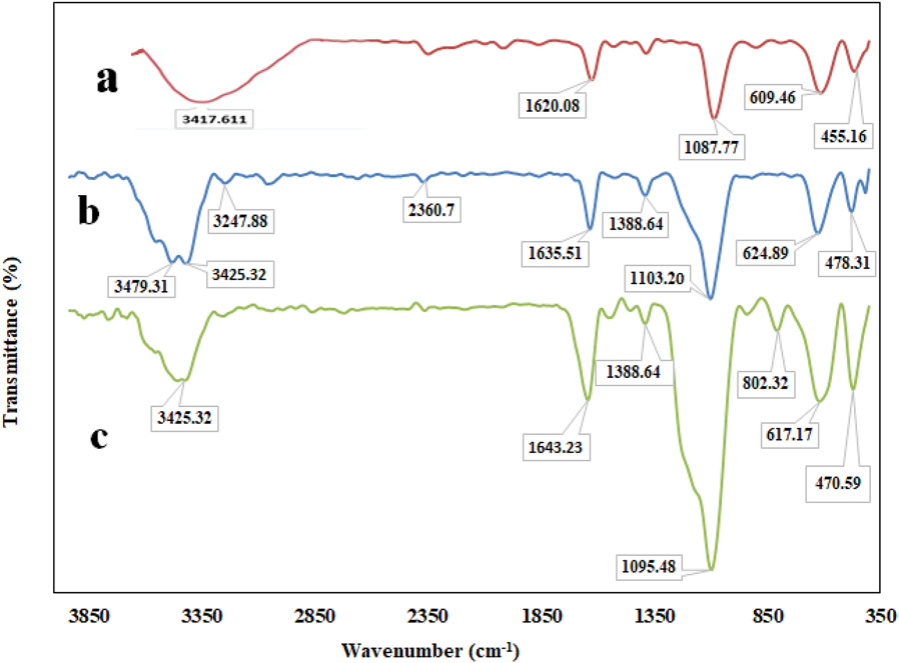
FT-IR spectra of **a** Fe3O4@SiO2 nanoparticles; **b** amine-functionalized magnetic nanoparticles (MNPs-NH2); and **c** immobilized derivative of TLL on magnetic nanoparticles (MNPs-TLL)

FT-IR spectroscopy was also used to confirm the successful functionalization of the support with amine groups. FT-IR spectra of Fe_3_O_4_@SiO_2_, MNPs-NH_2_ and the lipase immobilized preparation are presented in Fig. 2a–c. In Fe_3_O_4_@SiO_2_ nanoparticles (Fig. 2a), the band at 1087.77 cm^−1^ corresponds to Si–O–Si symmetric stretching vibrations, is an indication of the presence of SiO_2_. Figure 2a shows the presence of Fe–O stretching vibration at 609.46 cm^−1^ and also O–H stretching vibration owing to the physically absorbed water and surface hydroxyl groups approximately at 3400 cm^−1^. Decrease in intensity of this band after functionalization together with appearing a doublet peak located at 3425.32 and 1635.51 cm^−1^ clearly confirmed successful functionalization of the support by APTMS (Fig. 2b). The IR bands responsible for the lipase immobilized on the magnetic nanoparticles were observed at 1643.23 cm^−1^ for amide I and at 1388.64 cm^−1^ for amide II, respectively (Miao et al. 2018; Fig. 2c).

**Fig. 3:**
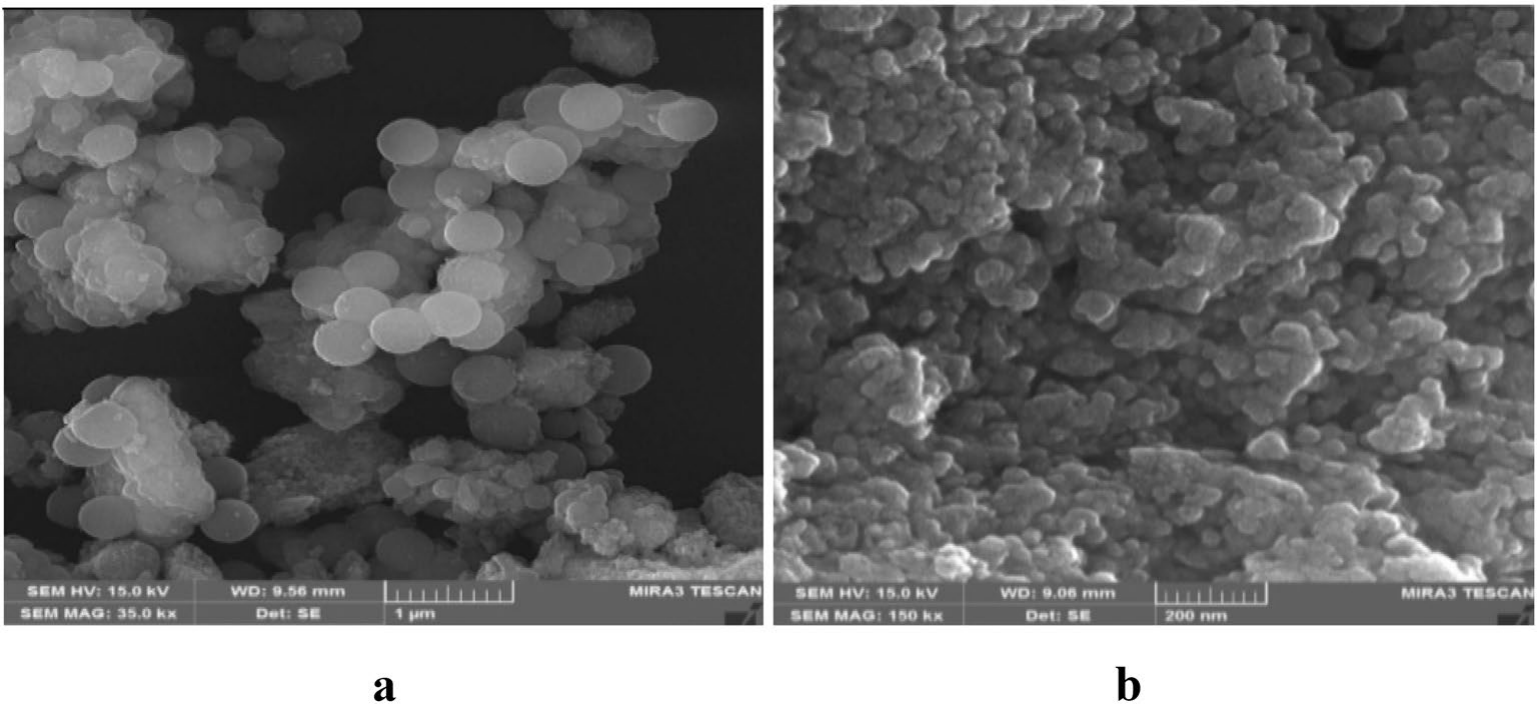
SEM images of **a** MNPs-NH_2_, and **b** MNPs-TLL

The morphology and structure of MNPs-NH_2_ and immobilized TLL were also characterized by SEM. Figure 3a and b indicates that samples consists of various bunches of spherical shaped in different sizes with uniform morphology distribution. By considering SEM images it can be concluded that the modification still preserving the textural properties of the MNPs.

### Immobilization of RML, TLL and CALB on MNPs-NH_2_

The approach of using isocyanide-based multi-component reaction provided a single-step, rapid and low-cost process for high-density covalent attachment of RML, TLL and CALB on amine-functionalized MNPs.‌ Immobilizations were carried out in water (pH 7.0) at 25 °C, in which high immobilization yields were obtained (92–100%). The leaching experiment was conducted to remove those enzyme molecules physically adsorbed on the support. Protein quantification in the washing solution was performed with both *p*-NPB assay and the Bradford method showing no detectable enzyme in the solution. This means that in the used immobilization method, the reaction between enzyme molecules and MNPs is exclusively carried out by covalent binding. Immobilization of lipases on MNPs-NH_2_ was investigated in terms of immobilization time, immobilization yield, loading capacity, and specific activity (Table 1). Various amounts of RML, TLL and CALB (20, 40, 60, 80, and 100 mg/g) was offered to 1 g of MNPs-NH_2_ under gentle stirring for up to 12 h. Immobilization of 20 mg of RML, TLL and CALB on 1 g of the support was carried out shortly after 2 h of incubation, producing 100%, 98.5%, and 99.2% immobilization yields, respectively. While we have already reported covalent immobilization of CALB on epoxy-functionalized Fe_3_O_4_@SiO_2_ within 24 h of incubation with 84% immobilization yield (Mehrasbi et al. 2017). The used strategy was based on traditional coupling process and resulted immobilization of 50 mg of CALB on 1 g of the support max. With increasing the amount of enzyme to 40 mg, almost the same immobilization yields were obtained with slight change in immobilization time of TLL and CALB (3 h). With further increasing the amount of suggested enzymes to 100 mg, the immobilization yields of 97.5%, 99.5%, and 94% were detected for RML, TLL and CALB, respectively, after 12 h of incubation. We have recently reported immobilization of RML and TLL on aldehyde-functionalized magnetic nanoparticles via multi-component reactions. Rapid immobilization processes within 12 h were observed producing 81% and 97% of immobilization yields for RML and TLL, respectively, by offering 100 mg of each enzyme per 1 g of the support. The maximum loading capacity of the aldehyde-functionalized support was determined to be 81 mg for RML and 97 mg for TLL which are lower than the obtained loading amount of TLL and RML in this study at the same conditions. Table 1 also shows specific activity of the free and immobilized enzymes. With increasing the amount of immobilized enzymes, lower specific activities were observed for the final biocatalysts. Decrease in enzyme specific activity during covalent immobilization has already been reported in several studies and has been mostly attributed to the enzyme denaturation caused during coupling process (Weltz et al. 2020). Since the specific activity of the immobilized derivatives obtained in immobilization with 20 mg of each lipase presented a small change in activity compared to the corresponding free enzyme, the remarkable decrease in activity in higher amounts of lipases can be attributed to the diffusion limitation of substrate/product in the immobilized preparations (Mohammadi et al. 2014). The activity recovery for the immobilized preparations was calculated as the ratio of the activity of immobilized enzyme to the activity of same amount of the corresponding soluble enzyme. The results showed that with increasing the amount of lipases loaded on the support the recovered activity decreased particularly for CALB with almost 65% lower recovered activity in 100 mg/g of offered enzyme compared to result obtained in the process for immobilization of 20 mg/g of the enzyme.

**Table 1:** Immobilization of the lipases on amine-functionalized magnetic nanoparticles

Offered enzyme (mg/g)	Yield (%)			Immobilized enzyme (mg/g support)			Immobilization time (h)			Specific activity U/mg (recovered activity)		
	RML	CALB	TLL	RML	CALB	TLL	RML	CALB	TLL	RML	CALB	TLL
20	100	99.2	98.5	20.0	19.8	19.7	2	2	2	8.2 (142)	35.0 (157)	26.8 (101)
40	100	98.5	98.7	40.0	39.4	39.5	2	3	3	7.5 (125)	30.1 (112)	23.4 (95)
60	98.5	95.0	98.3	59.1	57.0	58.9	3	5	5	6.6 (90)	24.6 (72)	18.3 (99)
80	96.0	92.32	93.7	76.8	73.8	75.0	12	12	12	5.3 (93)	18.9 (74)	13.7 (100)
100	97.5	94.0	99.5	97.5	94.0	99.5	12	12	12	4.2 (77)	9.3 (56)	9.8 (70)

The specific activities for the free forms of RML, CALB and TLL were 10.1 U/mg, 38.5 U/mg, and 27.5 U/mg, respectively

### Thermal stability of free and immobilized lipases

**Fig. 4:**
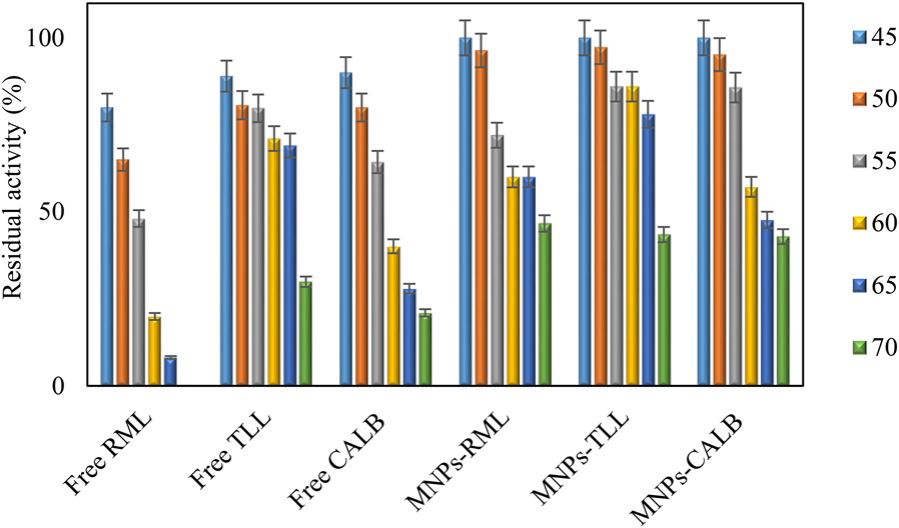
Effect of temperature on the activity of free and immobilized lipases. The incubation at different temperatures was carried out in 25 mM sodium phosphate buffer, pH 7.0. The initial activity of each derivative before incubation was determined and set as 100%

It is well documented that immobilization of enzymes can improve their thermal stability. Figure 4 represents the time course of residual activities of the free lipases and their immobilized derivatives at different temperatures up to 70 °C. At the lowest temperature tested (45 °C), the immobilized lipases on amine-functionalized MNPs remained completely active. While the free RML was relatively unstable and lost 40% of its initial activity at 50 °C, the soluble TLL and CALB showed more stability with retaining 80% of their activities. The immobilized forms of all enzymes remained 90% active at the same condition. At 55 °C, the immobilized RML showed 70% residual activity after 2 h of incubation, whereas, the soluble enzyme at the same temperature exhibited rapid inactivation. Increasing temperature to 60 °C showed 20%, 40% and 70% residual activity of free RML, CALB and TLL, respectively. Remaining 60% activity of MNPs-RML, MNPs-CALB and 80% activity of MNPs-TLL after 2 h incubation at 60 °C, confirmed the positive effect of immobilization on the stability of the lipases. With raising the temperature to 70 °C, the free RML completely lost its activity, while its immobilized form retained 45% of its initial activity. Bearing in mind the important role of aspartic acid residues on the surface of proteins, the higher stability of TLL derivative can be attributed to the higher number of exposed Asp residues on the surface of TLL (13) compared to RML (10) and CALB (9; Orrego et al. 2018).

### Co-solvent stability of the immobilized and free lipases

**Fig. 5:**
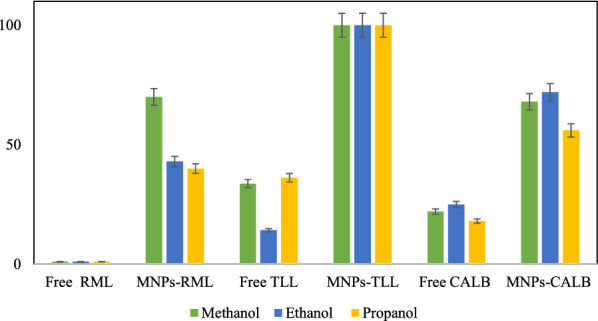
The stability of free and MNPs-RML, MNPs-TLL and MNPs-CALB in the presence of 50% of methanol, ethanol and propanol. Incubation of each biocatalyst in 1 ml solution containing 25 mM sodium phosphate buffer (pH 7.0) and 50% of each solvent at 25 °C. The initial activity of each derivative was determined before incubation and set as 100%

In the most enzymatic reactions, the use of co-solvent is essential to enhance the solubility of the organic substrates which are not soluble in water. To evaluate the stability of free and immobilized preparations of RML, TLL, and CALB, 5 mg of the derivatives (60 mg/g) were incubated for 24 h in the presence of 10%, 20%, and 50% of organic solvents including methanol, ethanol and 1-propanol (Fig. 5 and Additional file 1: Fig. S1a–c). The results could be interpreted by the effect of the carbon chain of each solvent on the activity of the enzymes. The results confirmed that using an organic solvent may influence the structure of the enzyme leads either to change the catalytic performance of the enzyme in desired enzymatic reaction or completely inactivation of the enzyme. This can be raised from the replacement of the crucial water molecules of enzyme structure (Nawani et al. 2006; Klibanov 1989) which are required for enzyme function. Organic solvents with log P < 2 have a propensity to strip this vital water and diminish the catalytic activity of enzyme (Klibanov 2001). Furthermore, enzymes usually tend to form aggregates in organic solvents which makes them poorly available for the substrate. Immobilization can remove this detrimental effect of organic solvents by individually fixing every single enzyme molecule separately on the support. Figure 5 shows the stability of the covalently immobilized preparations compared to the free lipases in the presence of 50% of three used co-solvents. The results showed that immobilized derivative of lipases retained their activity (over 70%) in the presence of 50% of methanol while the free lipases lost their activities in the same condition. In the presence of 10% and 20% ethanol, there were no significant differences between covalently immobilized lipases and free enzymes. With increasing the percentage of ethanol to 50%, while immobilized RML and CALB kept 43% and 72% of their activities, respectively, free CALB remained only 25% active and free RML was completely deactivated.

### Optimal pH activity of the immobilized preparations

**Fig. 6:**
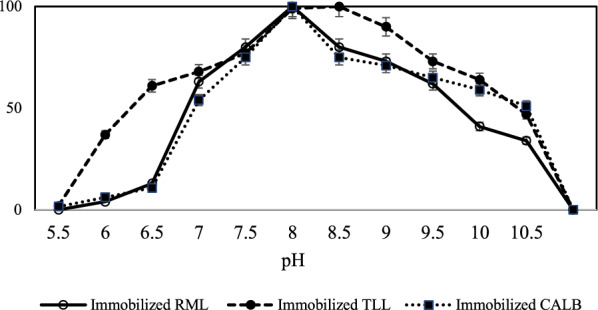
Effect of pH on activity of immobilized lipases

The activity of immobilized lipases at pH range between 5.5 and 10.5 was also investigated. As illustrated in Fig. 6, the maximum activity was observed at pH 8.0 and 8.5 for immobilized lipases. It was concluded that both lower and higher pH are unfavorable for the enzymatic activity of the lipases. This could be explained by the changes in the enzyme structure and ionic states of the active site residues. They also showed increased optimal pH activity compared to the corresponding free enzymes which have been reported to be 7.5 (Online et al. 2016; Bi et al. 2020; Mehrasbi et al. 2017). Furthermore, the immobilized TLL presented broader optimal pH adaptability ranging from 8 to 8.5.

### Biodiesel production

#### Optimization of the reaction conditions

In the present work, five factors such as (A) reaction temperature, (B) amount of water-adsorbent (RML and CALB) or water (TLL), (C) the amount of enzyme, (D) *t*-butanol weight percentage and (E) oil: methanol molar ratios were considered as effective parameters in biodiesel production. The yields of waste cooking oil methyl ester were from 30–61% for immobilized RML, 13–62% for immobilized TLL and 27–52% for immobilized CALB. Process optimization with response surface methodology (RSM) was performed and the effect of each factor and its interactions were calculated to ascertain the optimum conditions for the reaction. Among the models that fitted to the response such as linear, two factors interaction (2FI), quadratic, and cubic polynomial, the two factors interaction (2FI) was selected as the best model. This two-factor interaction (2FI) model was suggested by the RSM software as shown in Table 2 for TLL (Additional file 1: Table S1 for RML and Additional file 1: Table S2 for CALB). This two-factor interaction (2FI) model expressed by Eq. (3) shows biodiesel yield (Y), reaction temperature (A), water absorbent (RML and CALB) or water (TLL) w/w (B), the enzyme weight percentage ratio (C), the *t-*butanol weight percentage (D) and oil/methanol molar ratios (E). A positive sign in front of the terms indicates the synergistic effect of the rise in FAME yield, while negative sign indicates the antagonistic effect (Babaki et al. 2017). The results at each point based on the central composite design (CCD) and their corresponding predicted values are presented in Additional file 1: Table S3 for immobilized RML, Additional file 1: Table S4 for immobilized TLL and Additional file 1: Table S5 for immobilized CALB.

3 $$ \begin{aligned} {\text{Y}}_{{{\text{RML}}}} & = + { 4}0.{11 } - { 1}.{\text{83 A }} + \, 0.{\text{24 B }} + \, 0.{\text{37 C }} + { 2}.{\text{42 D }} \\ & \quad + \, 0.{\text{92 E }} - { 3}.{\text{22 AB }} - { 1}.{\text{88 AC }} + { 1}.0{\text{8 AD }} + {1}.{\text{58 AE }} \\ \quad + {2}.{\text{54 BC }} - \, 0.{\text{69 BD }} - { 2}.{\text{59 BE }} - { 2}.{\text{23 CD }} - \, 0.0{\text{42 CE }} + \, 0.{\text{49 DE}} \\ {\text{Y}}_{{{\text{TLL}}}} & = + { 37}.{97 } + { 2}.{\text{97A }}{-}{ 2}.{\text{42B }} + { 3}.{\text{95C }} + { 1}.{\text{13D }} + { 4}.{\text{14E }} \\ & \quad + \, 0.{\text{51AB }} - \, 0.{\text{34AC }} + { 2}.{\text{57 AD }} + 0.{\text{84 AE }} + { 1}.{8}0{\text{ BC }} + { 2}.0{\text{9BD }} - { 3}.{\text{42BE}} \\ \, & \quad - { 6}.{\text{86CD }} - { 1}.{4}0{\text{CE }}{-} \, 0.{\text{56DE}} \\ {\text{Y}}_{{{\text{CALB}}}} & = + { 4}0.{3}0 \, - { 1}.{\text{71A }} + { 1}.{2}0{\text{B }} - { 1}.00{\text{C }} - \, 0.{\text{75D }} - { 3}.{\text{63AB }} - { 1}.0{\text{8AD }} \\ & \quad + { 2}.{\text{97AE }} - \, 0.{\text{86BC }} - { 1}.{\text{76BD }} + {2}.0{\text{4BE }} - \, 0.{\text{91CD }} - {1}.{\text{94CE }} - {1}.{\text{83DE}}. \\ \end{aligned} $$

**Table 2:** Sequential model sum of squares for immobilized TLL

Source	Sum of squares	df	Mean square	*F* value	Prob > *F*	
Mean vs total	64,883.04	1	64,883.04			
Linear vs mean	1904.72	5	380.94	4.37	0.003	
2FI vs linear	2441.60	10	244.16	7.42	< 0.0001	Suggested
Quadratic vs 2FI	185.30	5	37.06	1.16	0.3591	
Cubic vs quadratic	660.28	15	44.02	3.63	0.0281	Aliased
Residual	109.06	9	12.12			
Total	70,184.00	45	1559.64			

The results of statistical analysis of variance (ANOVA) which were applied to determine the significance and fitness of the two factors interaction (2FI) model and also the effect of significant individual terms and their interaction on the selected responses for RML are presented in Additional file 1: Tables S6, S7 for TLL and Additional file 1: Table S8 for CALB. The *p* values were used as a tool to check the importance of each of the coefficients, which in turn may indicate the pattern of the interaction between the variables. Values of probability (*p*) > F less than 0.05 emphasized that the model terms were significant whereas the values greater than 0.05 indicate that the model terms were not significant (Chen et al. 2008). The terms incorporated in the model F-values of 5.79 for the immobilized RML, 8.80 for the immobilized TLL and 8.90 for the immobilized CALB with *p*-value < 0.0001 revealed that the model was significant at 95% confidence level. The *R*^2^ value of 0.74 for RML, 0.81 for TLL and 0.78 for CALB as well as adjusted *R*^2^ values of 0.62 for RML, 0.72 for TLL and 0.70 for CALB showed that the model was significant to predict the response. The predicted *R*^2^ values of 0.30 for RML, 0.53 for TLL and 0.62 for CALB were in reasonable agreement with the adjusted *R*^2^ values. The model also depicted the statistically non-significant lack of fit (p 0.39 for RML, p 0.46 for TLL and p 0.95 for CALB) which were not significant (*p*-value > 0.05 is not significant), indicating that the responses were adequate for utilizing in the model (Additional file 1: Fig. S2a for RML, Additional file 1: Fig. S2(b) for TLL and Additional file 1: Fig. S2(c) for CALB). These results also demonstrated that the model satisfactorily fitted to experimental data. Insignificant lack of fit was considered as the significant lack of fit indicating that there might be contribution in the regressor–response relationship that is not accounted for by the model (Noordin et al. 2004). This analysis was examined using the normal probability of the residuals (Additional file 1: Fig. S3a–c for TLL, RML and CALB, respectively). The normal probability plot of the residuals for RML, TLL and CALB showed that the errors were distributed normally in a straight line and insignificant. On the other hand, the plot of residuals versus predicted response indicated a structureless plot suggesting that the model was adequate and that they did not show any violation of the independence or constant variance assumption (Lee et al. 2011).

#### Effect of the reaction parameters on FAME yield

The effect of 5 parameters on the FAME yield was investigated for the immobilized lipases. MNPs-TLL was the only derivative that was affected by the reaction temperature and produced higher FAME yield with increasing the temperature. It has been already reported that, improved thermal stability after immobilization and decreasing mass transfer limitation between the reactants can be considered as important parameters to facilitate FAME production (Guo et al. 2020). The negative effect in fatty acid methyl esters yields was observed by increasing the water for MNPs-TLL, while for MNPs-CALB showed improved efficiency of biodiesel production. Zaks and coworkers reported that, the presence of excess water during biodiesel production might lead to aggregation of the enzyme and thus reducing its catalytic activity (Zaks and Klibanov 1988). The results also showed that increasing the amount of MNPs-RML had no effect on biodiesel production while it was effectively improved as the amount of MNPs-TLL increased (Tavares et al. 2017). The *t*-butanol percentage had negligible effect on biodiesel content for MNPs-TLL while the efficiency of FAME production was improved for MNPs-RML by providing higher contents of *t*-butanol. Change in the methanol:oil molar ratio showed different results for the immobilized derivatives. Increasing the methanol-to-oil ratio from 1:4 to 1:6 didn’t change the reaction yield for MNPs-RML while improved yield of FAME production was observed for MNPs-TLL.

**Fig. 7:**
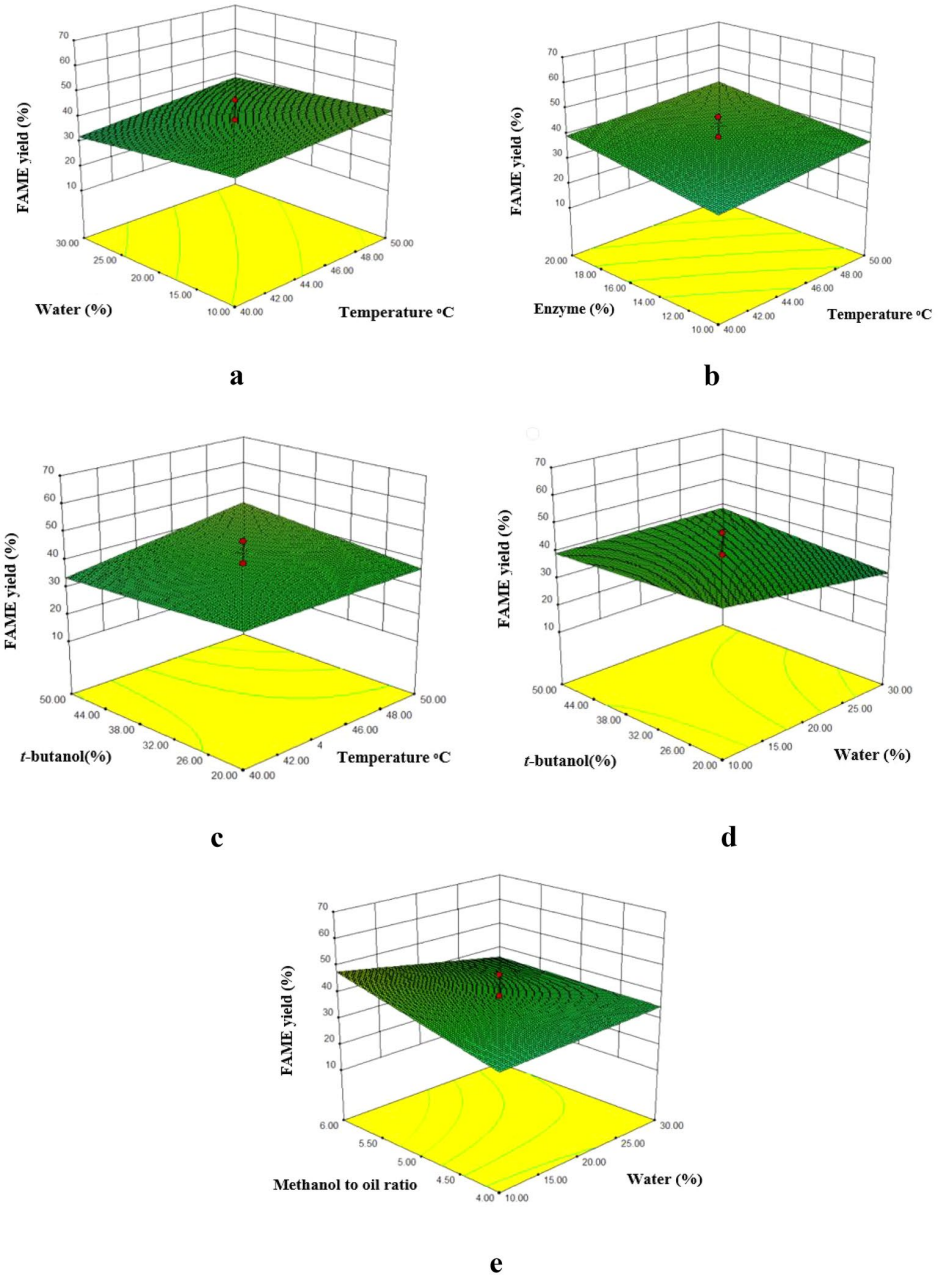
Response surface curves showing the interactions for MNPs-TLL. **a** Water content vs. temperature; **b** biocatalyst quantity vs. temperature; **c** *t*-butanol vs. temperature; **d** *t*-butanol vs. water content; **e** methanol-to-oil ratio vs. water content

The interaction of the selected variables and their effect on fatty acid methyl ester production were also evaluated for the immobilized TLL in Fig. 7 (Additional file 1: Fig. S4 for RML and Additional file 1: Fig. S5 for CALB). Although with increasing temperature the FAME yield slightly increased For MNPs-TLL, the response surface showed no improvement in FAME content in both levels of water (Fig. 7a). The interaction of reaction temperature and amount of biocatalyst for MNPs-TLL showed that with the simultaneous increase of temperature and amount of biocatalyst, the reaction efficiency improved (Fig. 7b)while for MNPs-RML the result was the opposite. The interaction effect between the temperature and *t*-butanol on biodiesel yield for MNPs-TLL showed that the FAME yield did not change with increasing temperature and keeping the amount of *t*-butanol constant (Fig. 7c), while with increasing temperature and *t*-butanol at the same time, the efficiency of biodiesel production increased significantly. The interaction between water content and *t*-butanol for MNPs-TLL showed that by increasing the amount of *t*-butanol and keeping the water constant (Fig. 7d), the yield was improved, while with increasing the amount of water and keeping the amount of *t*-butanol constant, the efficiency decreased significantly. For MNPs-TLL and MNPs-RML by increasing the amount of TLL and RML in a constant amount of *t*-butanol, FAME production was increased but for MNPs-CALB, with increasing the amount of biocatalyst there was no change in FAME yield. Furthermore, the simultaneous increase in the amount of biocatalyst and *t*-butanol reduced the FAME yield for all three enzymes. The interaction between water and methanol-to-oil ratio for MNPs-TLL showed that in a low amount of water (10%), increasing methanol concentration (methanol-to-oil ratio from 4:1 up to 6:1) resulted in higher FAME production (Fig. 7e), whereas at a high level of water (30%) the presence of higher methanol-to-oil ratio 6:1 caused to decrease in FAME yield. This is most likely because of the fact that with increasing methanol, the production of methyl ester first improved and then declined as a consequence of decrease in enzyme activity caused by excessive methanol (Yadav and Pawar 2012; Li et al. 2012). The effect of *t*-butanol and methanol-to-oil ratio for MNPs-TLL and MNPs-RML showed that the interaction of both low (20%) and high (50%) level of *t*-butanol with methanol-to-oil ratio could diminish the destructive effect of methanol on biocatalyst deactivation (Babaki et al. 2016). The negative effect of methanol on biodiesel production has been already reported particularly in high concentrations (Mohammadi et al. 2015). Different reports can be found on the use of lipases from *Thermomyces lanuginosus* lipase, *Candida antarctica* lipase B and *Rhizomucor miehei* lipase in transesterification of oils. The different results of each report is due to the fact that the immobilization protocol can effectively alter the catalytic performance and stability of each individual enzyme (Yousefi et al. 2020). As an example, Ashjari et al. have reported transesterification of waste cooking oil by using the immobilized RML on aldehyde-functionalized support that producing FAME yield of 57.5% (Ashjari et al. 2020b). The other research has showed encapsulation of RML in X-shaped zeolitic frameworks and use this biocatalyst to produce biodiesel with a conversion yield of 95.6% (Adnan et al. 2018). In addition, Cazaban et al. investigated biodiesel production from canola oil by using TLL immobilized on silica nanoparticles producing the maximum FAME yield of 88% (Cazaban et al. 2018).

### Reusability of the immobilized derivatives

**Fig. 8:**
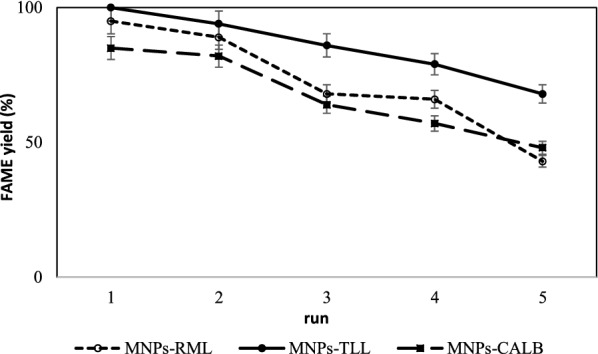
The effect of repeated use of the immobilized preparations on their activities in biodiesel production at 50 °C

The main advantage of immobilization is the reusability of the immobilized enzyme which decreases the final cost of the process. The immobilized derivatives of RML, TLL and CALB were investigated in terms of their operational stability in biodiesel production for 5 cycles of successive transesterification processes. After each run, the immobilized lipase was recovered, washed with n-hexane and dried to use in the next batch. The results showed that the derivatives have good capability to be repeatedly used up to five cycles with 43%, 68% and 48% of activity for MNPs-RML, MNPs-TLL, and MNPs-CALB, respectively (Fig. 8).

## Conclusion

This paper has investigated a facile method for the immobilization of lipases on amine-functionalized supports. This methodology is based on a three-component reaction including an amine from support, a carboxylic acid from enzyme surface and cyclohexyl isocyanide. RML, TLL and CALB were successfully immobilized on amine-functionalized magnetic nanoparticles at extremely mild conditions in a relatively short time. The leaching experiment confirmed that the enzymes were covalently attached on the support. The immobilized lipases were more active and stable in wide ranges of pH, organic solvents and different temperatures compared to their corresponding free forms. MNPs-TLL shows higher stability in comparison to MNPs-RML and MNPs-CALB, retaining 100% of its activity after 24 h of incubation in the presence of organic solvents. MNPs-TLL also showed broad range of optimum pH activity compared to its soluble form and the other immobilized derivatives. The immobilized lipases were then employed in biodiesel production by transesterification of waste cooking oil with methanol. In an optimization study, the effect of enzyme weight percentage ratio, *t*-butanol, temperature, methanol: oil ratio, water (for TLL) and the water-adsorbent weight percentage (for CALB and RML) on the FAME yield were evaluated. The maximal conversion to methyl esters of 78% was attained by using MNPs-CALB as catalyst and FAME production yield by immobilized TLL reached 81% under optimal conditions while the maximum yield for RML was 74%. Reusability study showed that the immobilized lipases on MNPs-NH_2_ can be easily recovered from the reaction mixture with retaining 43–68% of the initial activities after 5 cycles of the reaction.

### Supplementary Information

**Additional file 1: Table S1.** Sequential model sum of squares (RML). **Table S2.** Sequential model sum of squares (CALB). **Table S3.** Experimental design for five-level five-factor surface response design on transesterification of waste cooking oil using immobilized RML. **Table S4.** Experimental design for five-level five-factor surface response design on transesterification of waste cooking oil using immobilized TLL. **Table S5.** Experimental design for five-level five-factor surface response design on transesterification and esterification of waste cooking oil using immobilized CALB. **Table S6.** Analysis of variance (ANOVA) for fitted quadratic polynominal model for optimization of transesterification parameters (RML). **Table S7.** Analysis of variance (ANOVA) for fitted quadratic polynominal model for optimization of transesterification parameters (TLL). **Table S8.** Analysis of variance (ANOVA) for fitted quadratic polynominal model for optimization of transesterification parameters (CALB). **Fig. S1.** Stability of immobilized derivatives MNPs-RML (a) and MNPs-TLL (b) in the presence of 10, 20 and 50% of various co-solvents. **Fig. S2.** Predicted fatty acid methyl ester yield versus experimental fatty acid methyl ester yield for MNPs-RML (a), MNPs-TLL (b) and MNPs-CALB(c). **Fig. S3.** Normal probability plots of residuals for (a) MNPs-TLL (b), MNPs-RML and MNPs-CALB(c). **Fig. S4.** Response surface curves showing the interactions for RML. **Fig. S5.** Response surface curves showing the interactions for CALB.

## Data Availability

All data generated or analyzed during this study are included in this published article and its additional information files.
