# Supplementary material for: A multi-component reaction for covalent immobilization of lipases on amine-functionalized magnetic nanoparticles: production of biodiesel from waste cooking oil
Source: Bioresour Bioprocess. 2022 May 30;9(1):60. doi: 10.1186/s40643-022-00552-0 (PMC10991503; doi:10.1186/s40643-022-00552-0)
Supplement: Supplementary file 1 — Additional file 1: Table S1. Sequential model sum of squares (RML). Table S2. Sequential model sum of squares (CALB). Table S3. Experimental design for five-level five-factor surface response design on transesterification of waste cooking oil using immobilized RML. Table S4. Experimental design for five-level five-factor surface response design on transesterification of waste cooking oil using immobilized TLL. Table S5. Experimental design for five-level five-factor surface response design on transesterification and esterification of waste cooking oil using immobilized CALB. Table S6. Analysis of variance (ANOVA) for fitted quadratic polynominal model for optimization of transesterification parameters (RML). Table S7. Analysis of variance (ANOVA) for fitted quadratic polynominal model for optimization of transesterification parameters (TLL). Table S8. Analysis of variance (ANOVA) for fitted quadratic polynominal model for optimization of transesterification parameters (CALB). Fig. S1. Stability of immobilized derivatives MNPs-RML (a) and MNPs-TLL (b) in the presence of 10, 20 and 50% of various co-solvents. Fig. S2. Predicted fatty acid methyl ester yield versus experimental fatty acid methyl ester yield for MNPs-RML (a), MNPs-TLL (b) and MNPs-CALB(c). Fig. S3. Normal probability plots of residuals for (a) MNPs-TLL (b), MNPs-RML and MNPs-CALB(c). Fig. S4. Response surface curves showing the interactions for RML. Fig. S5. Response surface curves showing the interactions for CALB. [file 40643_2022_552_MOESM1_ESM.docx]

**A multi-component reaction for covalent immobilization of lipases on amine-functionalized magnetic nanoparticles: production of biodiesel from waste cooking oil**

Yalda Amini^a^, Zohreh Habibi ^a^*, Maryam Yousefi ^b^*, Maryam Ashjari ^a,c^, Mehdi Mohammadi ^c^, Mansour Shahedi^a^

^a^ Department of Organic Chemistry and Oil, Faculty of Chemistry, Shahid Beheshti University, Tehran, Iran

^b^ Nanobiotechnology Research Center, Avicenna Research Institute, ACECR, Tehran, Iran

^c^ Bioprocess Engineering Department, Institute of Industrial and Environmental Biotechnology, National Institute of Genetic Engineering and Biotechnology (NIGEB), Tehran, Iran

Table 1S Sequential Model Sum of Squares (RML)

| Source | Sum of Squares | df | Mean  Square | F value | Prob > F |  |
| --- | --- | --- | --- | --- | --- | --- |
| Mean vs Total | 72404.28 | 1 | 72404.28 |  |  |  |
| Linear vs Mean | 410.11 | 5 | 82.02 | 1.89 | 0.1181 |  |
| 2FI vs Linear | 1165.36 | 10 | 116.54 | 6.42 | < 0.0001 | Suggested |
| Quadratic vs 2FI | 70.70 | 5 | 14.14 | 0.75 | 0.5975 |  |
| Cubic vs Quadratic | 167.98 | 15 | 11.20 | 0.35 | 0.9647 | Aliased |
| Residual | 287.42 | 9 | 31.94 |  |  |  |
| Total | 74506.12 | 45 | 1655.69 |  |  |  |

Table 2S Sequential Model Sum of Squares (CALB)

| Source | Sum of Squares | df | Mean  Square | F value | Prob > F |  |
| --- | --- | --- | --- | --- | --- | --- |
| Mean vs Total | 73071.74 | 1 | 73071.74 |  |  |  |
| Linear vs Mean | 237.87 | 5 | 47.57 | 1.13 | 0.3627 |  |
| 2FI vs Linear | 1249.81 | 10 | 124.98 | 9.12 | < 0.0001 | Suggested |
| Quadratic vs 2FI | 113.08 | 5 | 22.62 | 1.91 | 0.1304 |  |
| Cubic vs Quadratic | 118.69 | 15 | 7.91 | 0.43 | 0.9288 | Aliased |
| Residual | 165.82 | 9 | 18.42 |  |  |  |
| Total | 74957.01 | 45 | 1665.71 |  |  |  |

Table 3S experimental design for five-level five-factor surface response design on transesterification of waste cooking oil using immobilized RML

| Run | A:Reaction | B:Water | C:Enzym | D:*t*-butanol | E:Methanol | FAME yield |
| --- | --- | --- | --- | --- | --- | --- |
|  | Temperature | absorbent |  |  | to oil | % |
| 1 | 40 | 50 | 20 | 20 | 4 | 59.9 |
| 2 | 40 | 50 | 20 | 50 | 4 | 47.1 |
| 3 | 55 | 40 | 15 | 35 | 5 | 32.1 |
| 4 | 35 | 40 | 15 | 35 | 5 | 40.8 |
| 5 | 40 | 30 | 20 | 50 | 4 | 42.5 |
| 6 | 45 | 40 | 15 | 35 | 3 | 35.8 |
| 7 | 40 | 50 | 10 | 50 | 6 | 42.3 |
| 8 | 40 | 50 | 10 | 20 | 6 | 30.6 |
| 9 | 50 | 50 | 10 | 50 | 4 | 39.8 |
| 10 | 45 | 40 | 15 | 35 | 5 | 40.9 |
| 11 | 45 | 60 | 15 | 35 | 5 | 37.8 |
| 12 | 50 | 50 | 20 | 50 | 4 | 39.2 |
| 13 | 40 | 30 | 10 | 20 | 4 | 35.3 |
| 14 | 45 | 40 | 15 | 35 | 7 | 43.6 |
| 15 | 50 | 30 | 10 | 50 | 6 | 60.9 |
| 16 | 50 | 30 | 20 | 50 | 6 | 41.5 |
| 17 | 50 | 50 | 10 | 50 | 6 | 39.1 |
| 18 | 50 | 30 | 10 | 20 | 4 | 34.9 |
| 19 | 40 | 30 | 20 | 20 | 6 | 44.2 |
| 20 | 50 | 30 | 10 | 50 | 4 | 47.6 |
| 21 | 45 | 40 | 15 | 35 | 5 | 35.8 |
| 22 | 50 | 30 | 20 | 50 | 4 | 33.1 |
| 23 | 40 | 30 | 10 | 50 | 4 | 42.1 |
| 24 | 45 | 40 | 5 | 35 | 5 | 33.1 |
| 25 | 45 | 40 | 25 | 35 | 5 | 42.5 |
| 26 | 40 | 30 | 10 | 50 | 6 | 47.5 |
| 27 | 50 | 30 | 10 | 20 | 6 | 41.9 |
| 28 | 50 | 50 | 20 | 20 | 6 | 30.6 |
| 29 | 50 | 50 | 20 | 50 | 6 | 39.8 |
| 30 | 40 | 50 | 20 | 20 | 6 | 47.9 |
| 31 | 40 | 30 | 10 | 20 | 6 | 37.8 |
| 32 | 45 | 40 | 15 | 35 | 5 | 35.2 |
| 33 | 50 | 50 | 20 | 20 | 4 | 35.3 |
| 34 | 50 | 30 | 20 | 20 | 6 | 43.6 |
| 35 | 40 | 50 | 10 | 50 | 4 | 45.9 |
| 36 | 45 | 40 | 15 | 5 | 5 | 32.7 |
| 37 | 40 | 50 | 20 | 50 | 6 | 49.1 |
| 38 | 50 | 30 | 20 | 20 | 4 | 34.9 |
| 39 | 45 | 40 | 15 | 65 | 5 | 44.2 |
| 40 | 50 | 50 | 10 | 20 | 6 | 34.6 |
| 41 | 50 | 50 | 10 | 20 | 4 | 31.2 |
| 42 | 40 | 30 | 20 | 50 | 6 | 35.4 |
| 43 | 45 | 20 | 15 | 35 | 5 | 35.8 |
| 44 | 40 | 50 | 10 | 20 | 4 | 46.5 |
| 45 | 40 | 30 | 20 | 20 | 4 | 30.2 |

Table 4S experimental design for five-level five-factor surface response design on transesterification of waste cooking oil using immobilized TLL

| Run | A: Reaction | B: Water | C: Enzyme | D: *t*-butanol | E: Methanol | FAME yield |
| --- | --- | --- | --- | --- | --- | --- |
|  | Temperature |  |  |  | to oil | % |
| 1 | 40 | 10 | 20 | 20 | 4 | 38.2 |
| 2 | 50 | 30 | 20 | 20 | 6 | 44.9 |
| 3 | 40 | 10 | 20 | 20 | 6 | 61.9 |
| 4 | 45 | 0 | 15 | 35 | 5 | 50.3 |
| 5 | 50 | 30 | 20 | 50 | 6 | 40.9 |
| 6 | 50 | 30 | 20 | 20 | 4 | 49.4 |
| 7 | 45 | 20 | 5 | 35 | 5 | 29.3 |
| 8 | 50 | 30 | 10 | 50 | 6 | 54.3 |
| 9 | 50 | 10 | 10 | 50 | 4 | 38.5 |
| 10 | 40 | 10 | 20 | 50 | 4 | 30.4 |
| 11 | 50 | 10 | 20 | 50 | 6 | 51.5 |
| 12 | 45 | 20 | 15 | 35 | 5 | 46.8 |
| 13 | 40 | 30 | 10 | 50 | 6 | 42.8 |
| 14 | 40 | 10 | 20 | 50 | 6 | 28.2 |
| 15 | 50 | 10 | 10 | 20 | 4 | 25.3 |
| 16 | 45 | 40 | 15 | 35 | 5 | 30.3 |
| 17 | 50 | 30 | 10 | 20 | 4 | 22.1 |
| 18 | 45 | 20 | 15 | 65 | 5 | 41.2 |
| 19 | 50 | 30 | 10 | 20 | 6 | 19.0 |
| 20 | 45 | 20 | 15 | 35 | 3 | 30.9 |
| 21 | 40 | 10 | 10 | 50 | 4 | 33.7 |
| 22 | 50 | 10 | 20 | 20 | 6 | 53.1 |
| 23 | 55 | 20 | 15 | 35 | 5 | 37.8 |
| 24 | 45 | 20 | 15 | 35 | 5 | 38.8 |
| 25 | 40 | 10 | 10 | 20 | 4 | 23.4 |
| 26 | 45 | 20 | 25 | 35 | 5 | 56.0 |
| 27 | 40 | 30 | 20 | 20 | 4 | 48.3 |
| 28 | 40 | 30 | 20 | 20 | 6 | 44.4 |
| 29 | 40 | 30 | 10 | 20 | 6 | 22.2 |
| 30 | 40 | 30 | 10 | 50 | 4 | 33.2 |
| 31 | 50 | 10 | 10 | 20 | 6 | 46.2 |
| 32 | 40 | 10 | 10 | 50 | 6 | 40.4 |
| 33 | 50 | 30 | 20 | 50 | 4 | 42.2 |
| 34 | 40 | 30 | 20 | 50 | 6 | 26.0 |
| 35 | 40 | 30 | 10 | 20 | 4 | 13.3 |
| 36 | 40 | 10 | 10 | 20 | 6 | 42.1 |
| 37 | 45 | 20 | 15 | 5 | 5 | 32.0 |
| 38 | 50 | 10 | 20 | 20 | 4 | 35.3 |
| 39 | 45 | 20 | 15 | 35 | 5 | 38.8 |
| 40 | 40 | 30 | 20 | 50 | 4 | 31.4 |
| 41 | 45 | 20 | 15 | 35 | 7 | 44.3 |
| 42 | 50 | 10 | 20 | 50 | 4 | 28.6 |
| 43 | 35 | 20 | 15 | 35 | 5 | 24.7 |
| 44 | 50 | 30 | 10 | 50 | 4 | 39.6 |
| 45 | 50 | 10 | 10 | 50 | 6 | 54.1 |

|  |  |  |  |  |  |  |
| --- | --- | --- | --- | --- | --- | --- |

Table 5S experimental design for five-level five-factor surface response design on transesterification and esterification of waste cooking oil using immobilized CALB

| Run | A:Reaction | B:Water | C:Enzyme | D:*t*-butanol | E:Methanol | FAME yield |
| --- | --- | --- | --- | --- | --- | --- |
|  | Temperature | absorbent |  |  | to oil | % |
| 1 | 40 | 50 | 20 | 20 | 4 | 49.0 |
| 2 | 40 | 50 | 20 | 50 | 4 | 49.0 |
| 3 | 55 | 40 | 15 | 35 | 5 | 35.6 |
| 4 | 35 | 40 | 15 | 35 | 5 | 34.1 |
| 5 | 40 | 30 | 20 | 50 | 4 | 42.4 |
| 6 | 45 | 40 | 15 | 35 | 3 | 43.0 |
| 7 | 40 | 50 | 10 | 50 | 6 | 48.9 |
| 8 | 40 | 50 | 10 | 20 | 6 | 52.2 |
| 9 | 50 | 50 | 10 | 50 | 4 | 28.6 |
| 10 | 45 | 40 | 15 | 35 | 5 | 50.6 |
| 11 | 45 | 60 | 15 | 35 | 5 | 38.1 |
| 12 | 50 | 50 | 20 | 50 | 4 | 27.6 |
| 13 | 40 | 30 | 10 | 20 | 4 | 35.3 |
| 14 | 45 | 40 | 15 | 35 | 7 | 44.5 |
| 15 | 50 | 30 | 10 | 50 | 6 | 39.4 |
| 16 | 50 | 30 | 20 | 50 | 6 | 31.9 |
| 17 | 50 | 50 | 10 | 50 | 6 | 41.4 |
| 18 | 50 | 30 | 10 | 20 | 4 | 34.0 |
| 19 | 40 | 30 | 20 | 20 | 6 | 29.0 |
| 20 | 50 | 30 | 10 | 50 | 4 | 41.5 |
| 21 | 45 | 40 | 15 | 35 | 5 | 39.6 |
| 22 | 50 | 30 | 20 | 50 | 4 | 45.4 |
| 23 | 40 | 30 | 10 | 50 | 4 | 47.1 |
| 24 | 45 | 40 | 5 | 35 | 5 | 41.0 |
| 25 | 45 | 40 | 25 | 35 | 5 | 40.3 |
| 26 | 40 | 30 | 10 | 50 | 6 | 36.4 |
| 27 | 50 | 30 | 10 | 20 | 6 | 45.9 |
| 28 | 50 | 50 | 20 | 20 | 6 | 40.9 |
| 29 | 50 | 50 | 20 | 50 | 6 | 33.0 |
| 30 | 40 | 50 | 20 | 20 | 6 | 47.1 |
| 31 | 40 | 30 | 10 | 20 | 6 | 32.0 |
| 32 | 45 | 40 | 15 | 35 | 5 | 40.3 |
| 33 | 50 | 50 | 20 | 20 | 4 | 34.1 |
| 34 | 50 | 30 | 20 | 20 | 6 | 44.3 |
| 35 | 40 | 50 | 10 | 50 | 4 | 49.0 |
| 36 | 45 | 40 | 15 | 5 | 5 | 42.4 |
| 37 | 40 | 50 | 20 | 50 | 6 | 36.0 |
| 38 | 50 | 30 | 20 | 20 | 4 | 37.5 |
| 39 | 45 | 40 | 15 | 65 | 5 | 38.7 |
| 40 | 50 | 50 | 10 | 20 | 6 | 49.4 |
| 41 | 50 | 50 | 10 | 20 | 4 | 31.4 |
| 42 | 40 | 30 | 20 | 50 | 6 | 33.1 |
| 43 | 45 | 20 | 15 | 35 | 5 | 38.6 |
| 44 | 40 | 50 | 10 | 20 | 4 | 49.0 |
| 45 | 40 | 30 | 20 | 20 | 4 | 42.4 |

Table 6S analysis of variance (ANOVA) for fitted quadratic polynominal model for optimization of transesterification parameters (RML)

|  | Sum of squares | df | Mean square | F value | p-value  Prob>F |  |
| --- | --- | --- | --- | --- | --- | --- |
| Model | 1575.47 | 15 | 105.03 | 5.79 | < 0.0001 | Significant |
| A-Temprature | 134.54 | 1 | 134.54 | 7.42 | 0.0108 |  |
| B-Water adsorbance | 2.28 | 1 | 2.28 | 0.13 | 0.7258 |  |
| C-Enzyme | 5.58 | 1 | 5.58 | 0.31 | 0.5834 |  |
| D-t-butanol | 233.87 | 1 | 233.87 | 12.89 | 0.0012 |  |
| E-Methanol to oil | 33.84 | 1 | 33.84 | 1.87 | 0.1825 |  |
| AB | 331.74 | 1 | 331.74 | 18.29 | 0.0002 |  |
| AC | 112.54 | 1 | 112.54 | 6.20 | 0.0187 |  |
| AD | 37.12 | 1 | 37.12 | 2.05 | 0.1633 |  |
| AE | 80.17 | 1 | 80.17 | 4.42 | 0.0443 |  |
| BC | 206.66 | 1 | 206.66 | 11.39 | 0.0021 |  |
| BD | 15.13 | 1 | 15.13 | 0.83 | 0.3687 |  |
| BE | 215.20 | 1 | 215.20 | 11.86 | 0.0018 |  |
| CD | 159.19 | 1 | 159.19 | 8.77 | 0.0060 |  |
| CE | 0.056 | 1 | 0.056 | 3.089E-003 | 0.9561 |  |
| DE | 7.54 | 1 | 7.54 | 0.42 | 0.5241 |  |
| Residual | 526.10 | 29 | 18.14 |  |  |  |
| Lack of Fit | 506.97 | 27 | 18.78 | 1.96 | 0.3935 | not significant |
| Pure Error | 19.13 | 2 | 9.56 |  |  |  |
| Cor Total | 2101.57 | 44 |  |  |  |  |

Table 7S. Analysis of variance (ANOVA) for fitted quadratic polynominal model for optimization of transesterification parameters (TLL)

|  | Sum of squares | df | Mean square | F value | p-value  Prob>F |  |
| --- | --- | --- | --- | --- | --- | --- |
| Model | 4346.32 | 15 | 289.75 | 8.80 | < 0.0001 | significant |
| A-Temprature | 310.02 | 1 | 310.02 | 9.42 | 0.0046 |  |
| B-Water | 234.49 | 1 | 234.49 | 7.12 | 0.0123 |  |
| C-Enzyme | 622.96 | 1 | 622.96 | 18.92 | 0.0002 |  |
| D-*t*-butanol | 50.77 | 1 | 50.77 | 1.54 | 0.2242 |  |
| E-Methanol to oil | 686.48 | 1 | 686.48 | 20.85 | < 0.0001 |  |
| AB | 8.31 | 1 | 8.31 | 0.25 | 0.6193 |  |
| AC | 3.64 | 1 | 3.64 | 0.11 | 0.7420 |  |
| AD | 210.88 | 1 | 210.88 | 6.41 | 0.0171 |  |
| AE | 22.40 | 1 | 22.40 | 0.68 | 0.4162 |  |
| BC | 104.41 | 1 | 104.41 | 3.17 | 0.0854 |  |
| BD | 140.39 | 1 | 140.39 | 4.26 | 0.0480 |  |
| BE | 374.62 | 1 | 374.62 | 11.37 | 0.0021 |  |
| CD | 1504.35 | 1 | 1504.35 | 45.70 | < 0.0001 |  |
| CE | 63.13 | 1 | 63.13 | 1.92 | 0.1767 |  |
| DE | 10.07 | 1 | 10.07 | 0.31 | 0.5844 |  |
| Residual | 954.64 | 29 | 32.92 |  |  |  |
| Lack of Fit | 911.82 | 27 | 33.77 | 1.58 | 0.4618 | not significant |
| Pure Error | 42.81 | 2 | 21.41 |  |  |  |
| Cor Total | 5300.96 | 44 |  |  |  |  |

Table 8S analysis of variance (ANOVA) for fitted quadratic polynominal model for optimization of transesterification parameters (CALB)

|  | Sum of squares | df | Mean square | F value | p-value  Prob>F |  |
| --- | --- | --- | --- | --- | --- | --- |
| Model | 1486.95 | 13 | 114.38 | 8.90 | < 0.0001 | significant |
| A-Temprature | 116.85 | 1 | 116.85 | 9.09 | 0.0051 |  |
| B-Water adsorbance | 58.01 | 1 | 58.01 | 4.51 | 0.0417 |  |
| C-Enzyme | 40.31 | 1 | 40.31 | 3.14 | 0.0864 |  |
| D-t-butanol | 22.70 | 1 | 22.70 | 1.77 | 0.1935 |  |
| AB | 421.55 | 1 | 421.55 | 32.81 | < 0.0001 |  |
| AD | 37.54 | 1 | 37.54 | 2.92 | 0.0974 |  |
| AE | 281.34 | 1 | 281.34 | 21.90 | < 0.0001 |  |
| BC | 23.65 | 1 | 23.65 | 1.84 | 0.1847 |  |
| BD | 98.91 | 1 | 98.91 | 7.70 | 0.0093 |  |
| BE | 132.87 | 1 | 132.87 | 10.34 | 0.0030 |  |
| CD | 26.29 | 1 | 26.29 | 2.05 | 0.1626 |  |
| CE | 119.94 | 1 | 119.94 | 9.33 | 0.0046 |  |
| DE | 107.00 | 1 | 107.00 | 8.33 | 0.0070 |  |
| Residual | 398.31 | 31 | 12.85 |  |  |  |
| Lack of Fit | 322.56 | 29 | 11.12 | 0.29 | 0.9531 | not significant |
| Pure Error | 75.76 | 2 | 37.88 |  |  |  |
| Cor Total | 1885.27 | 44 |  |  |  |  |

a

b

Fig.1S. Stability of immobilized derivatives MNPs-RML (a) and MNPs-TLL (b) in the presence of 10, 20 and 50% of various co-solvents


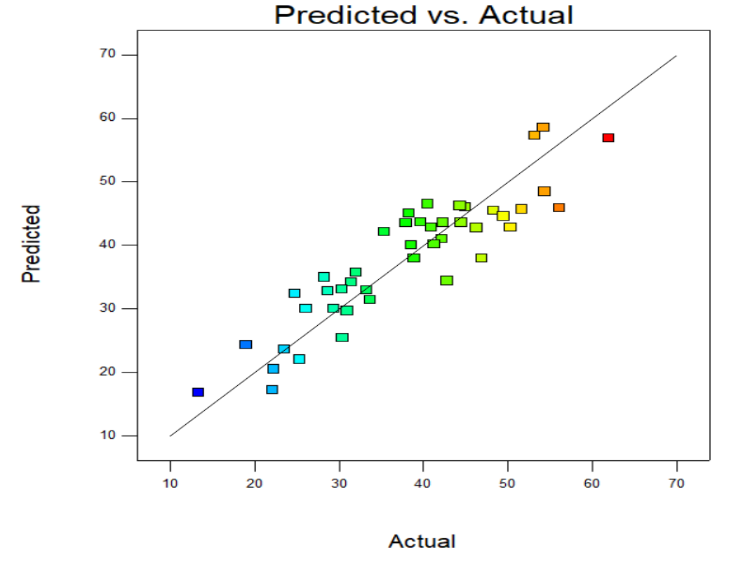

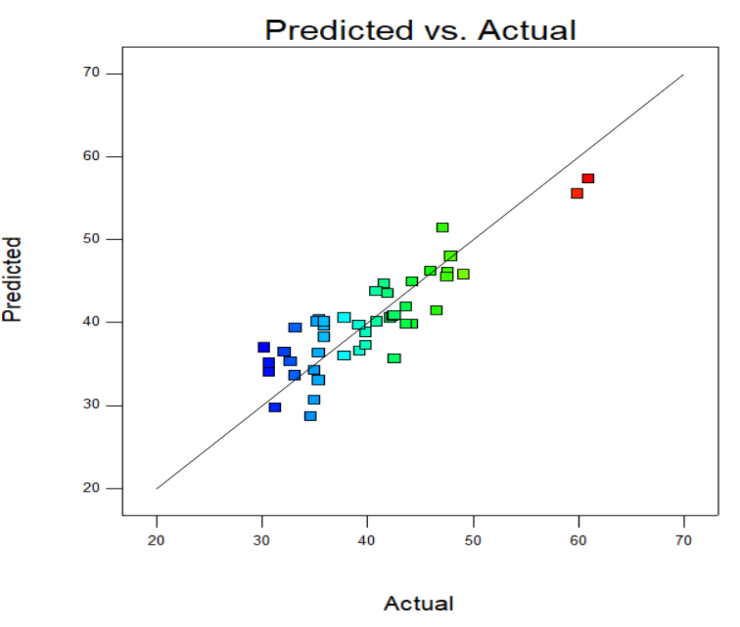


a b


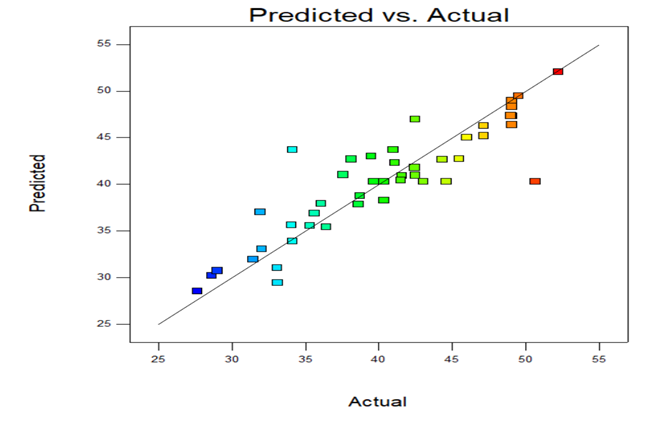


c

Fig.2S. Predicted fatty acid methyl ester yield versus experimental fatty acid methyl ester yield for MNPs-RML (a), MNPs-TLL (b) and MNPs-CALB(c)


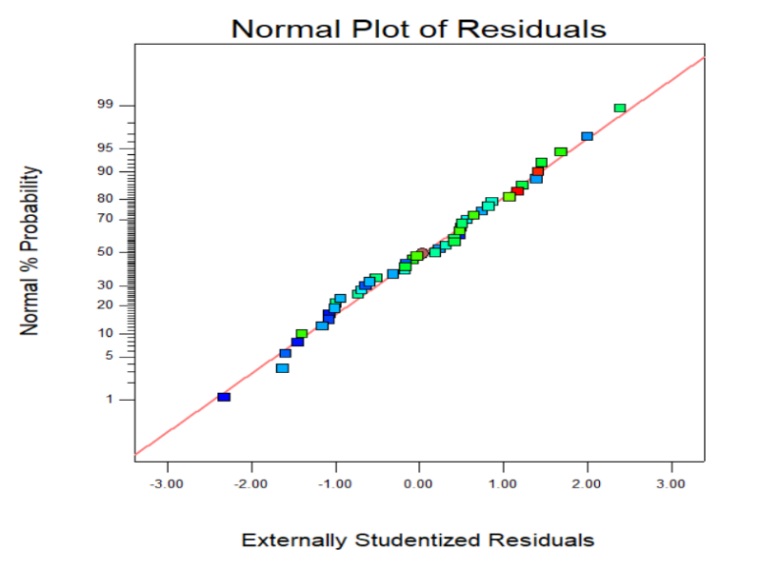

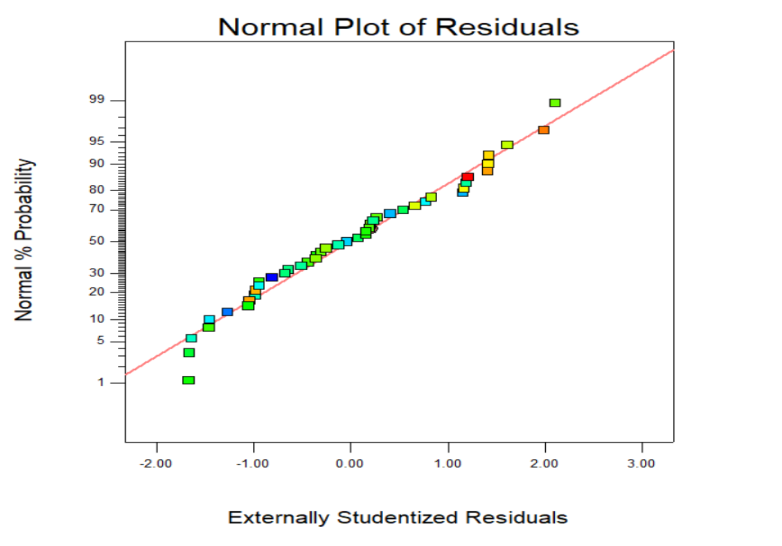


a b

**
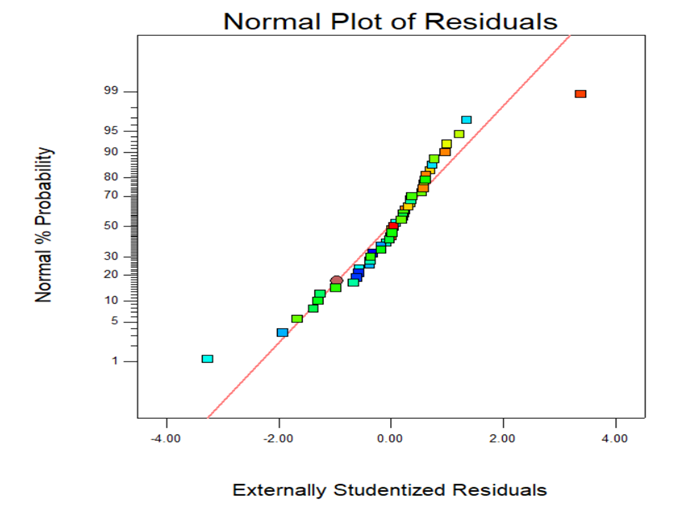
**

c

Fig.3S. Normal probability plots of residuals for (a) MNPs-TLL (b), MNPs-RML and MNPs-CALB(c).


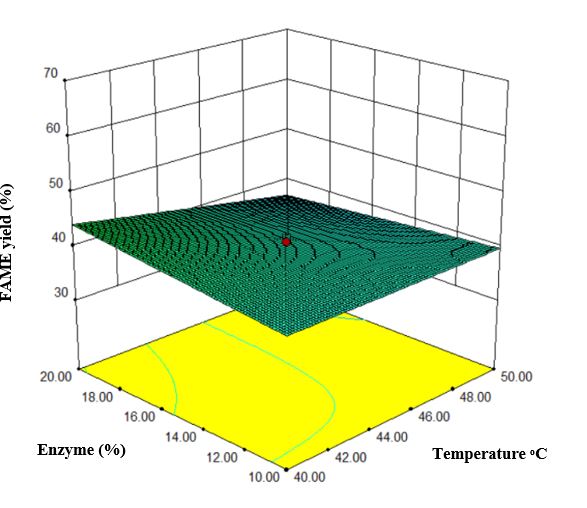
**
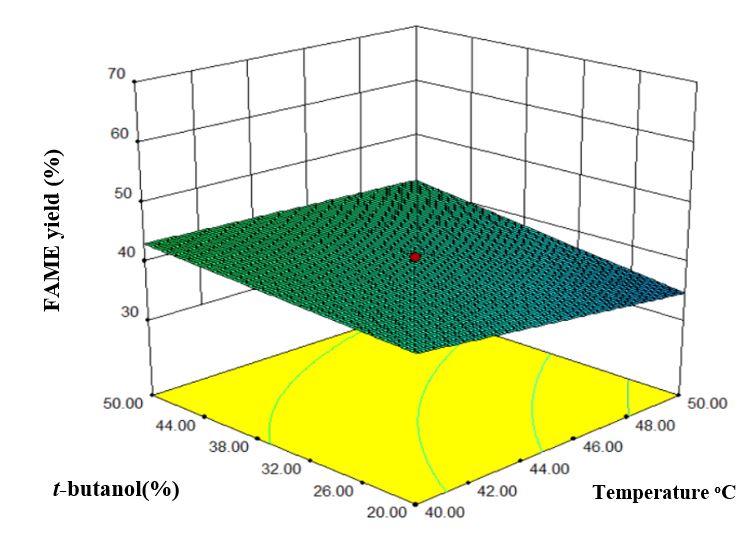
**


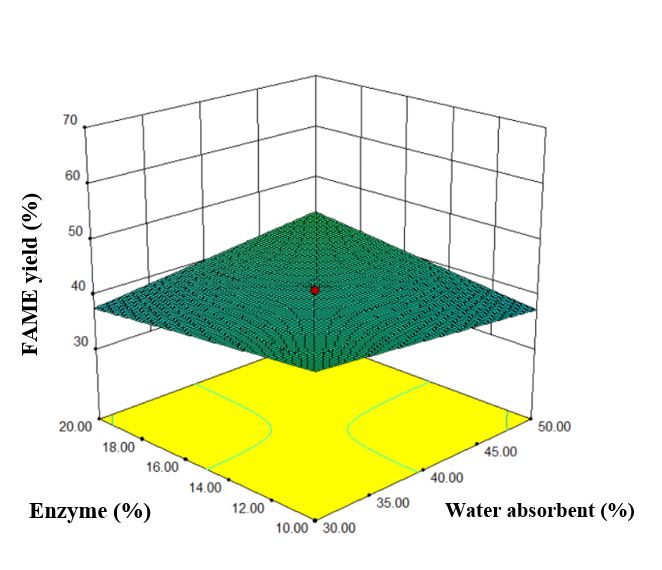
**
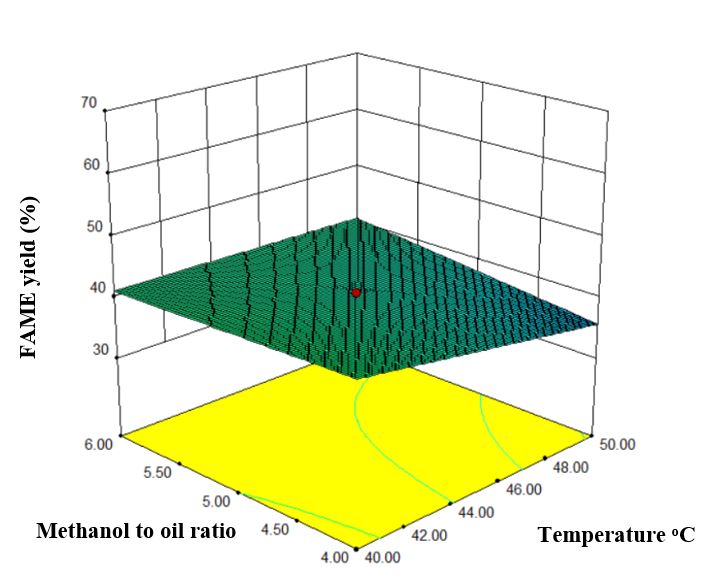
**


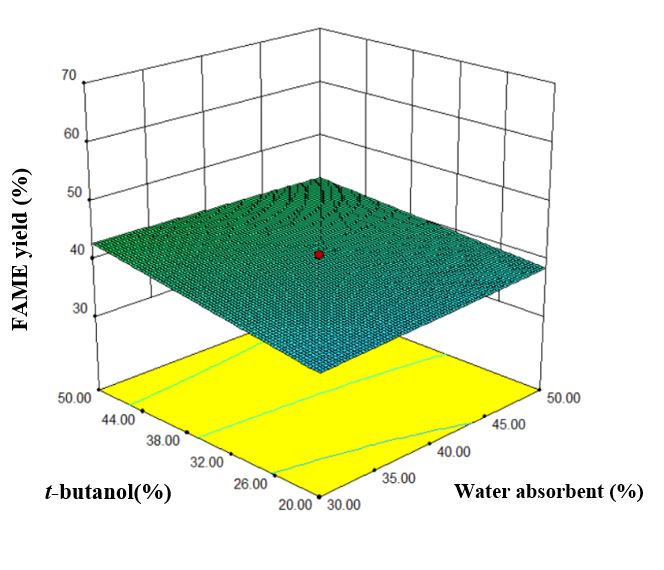

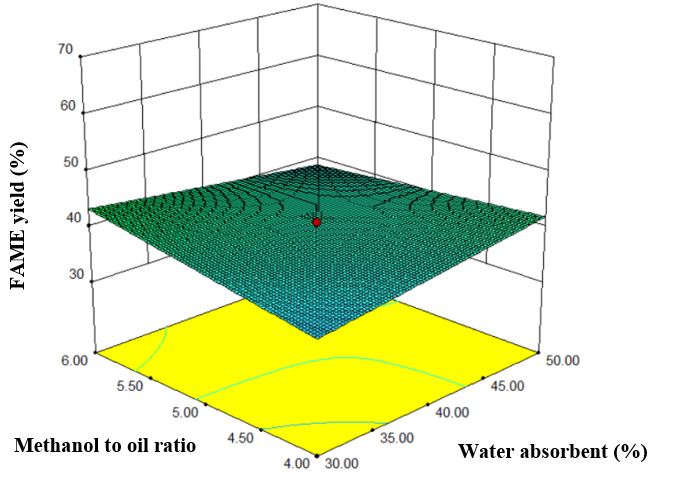


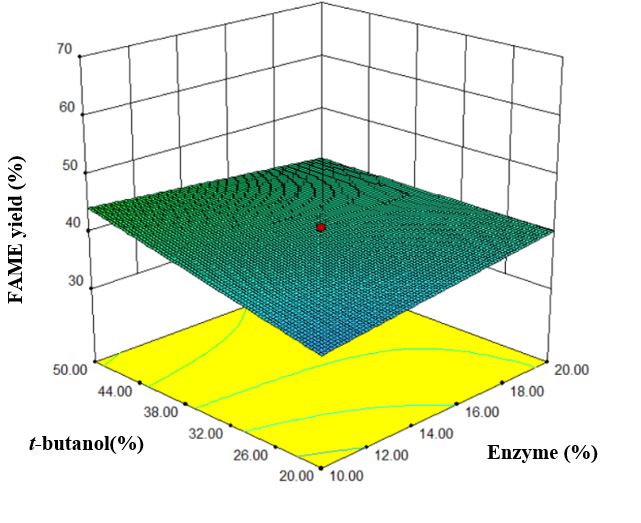

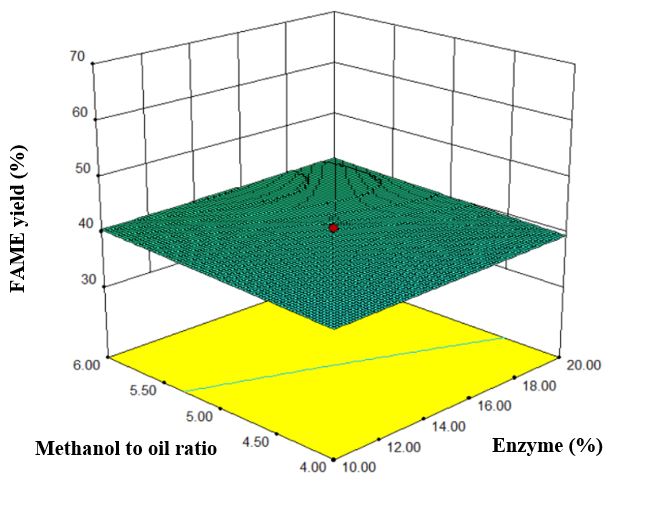


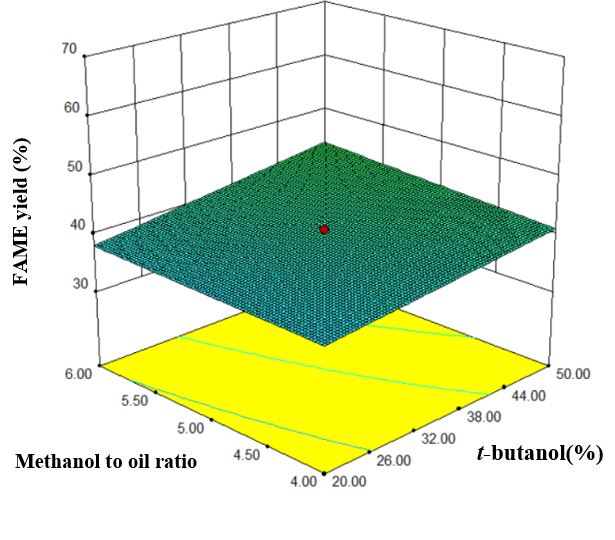


Figure 4S. Response surface curves showing the interactions for RML


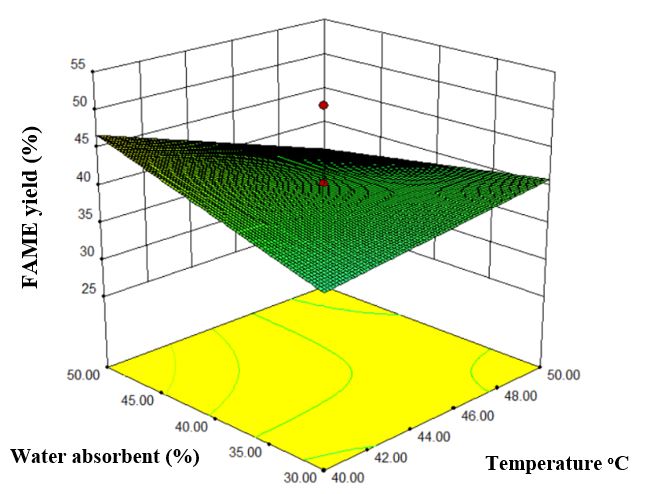

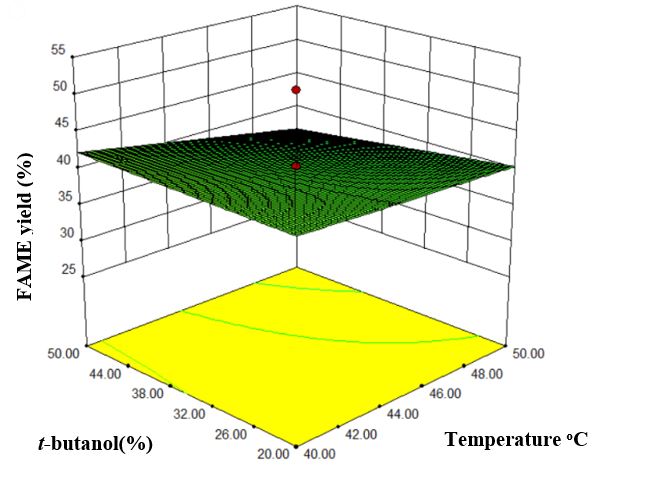


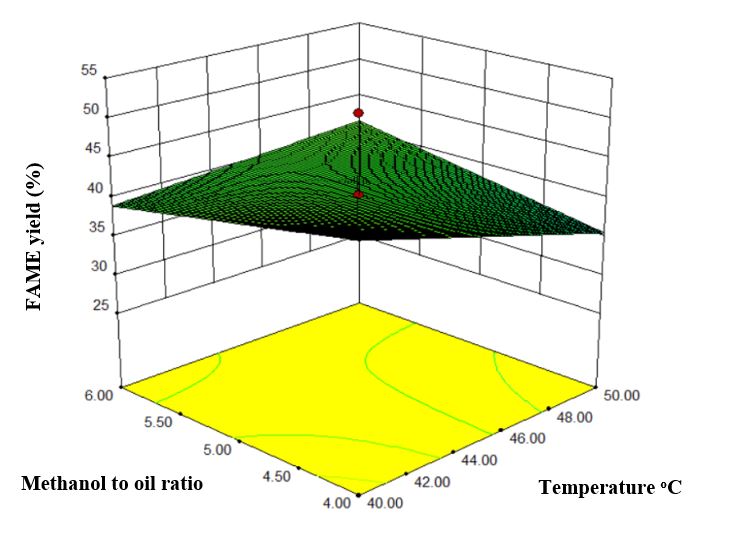

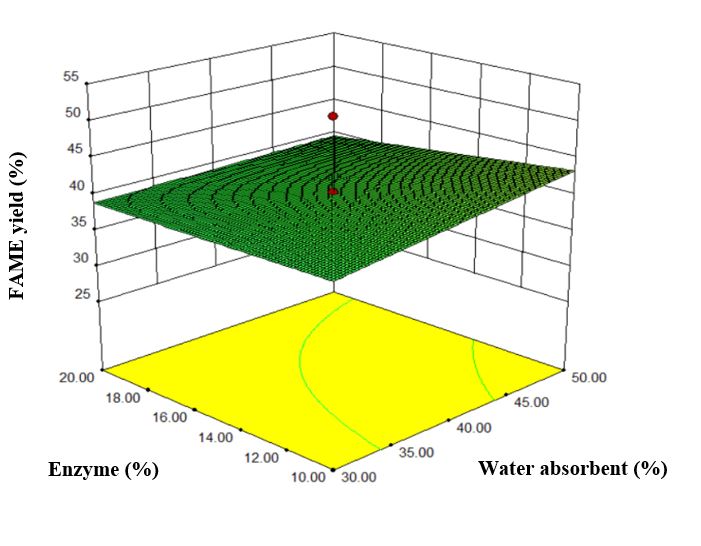


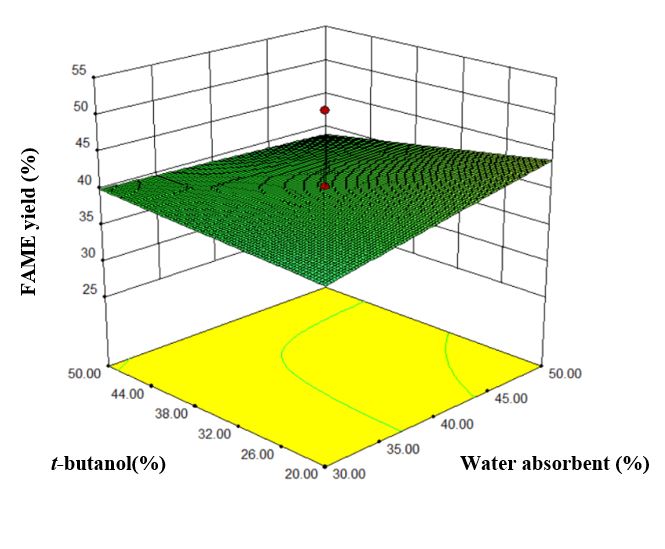


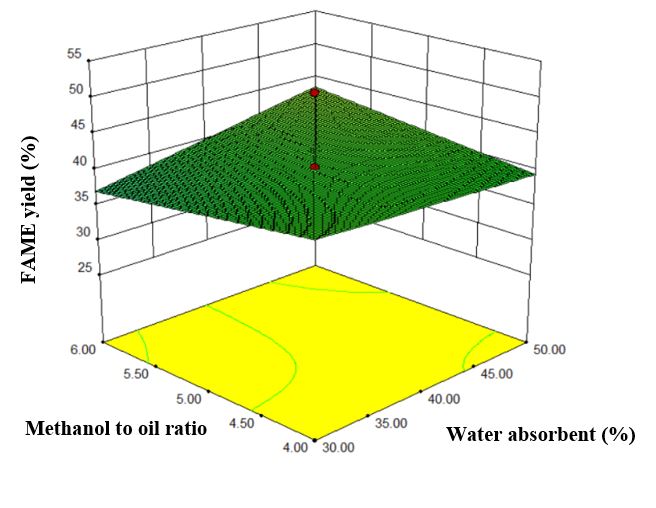


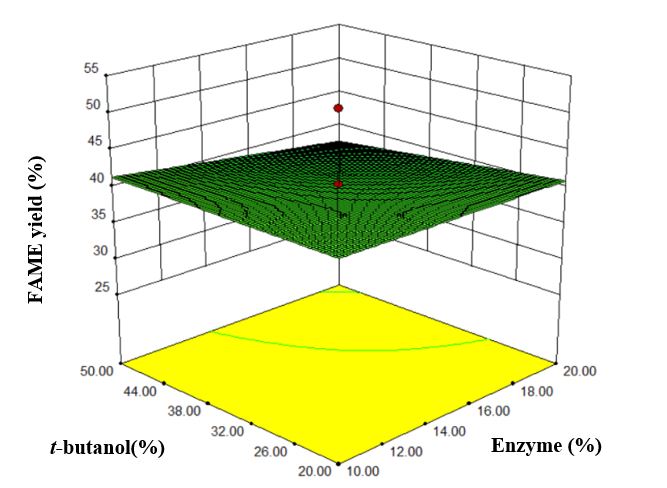

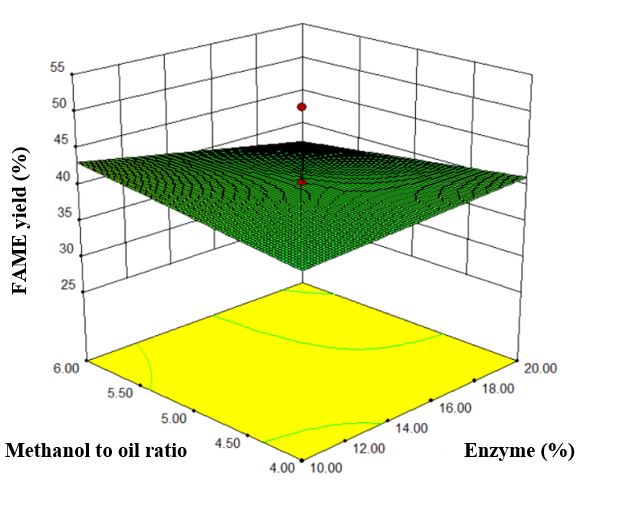


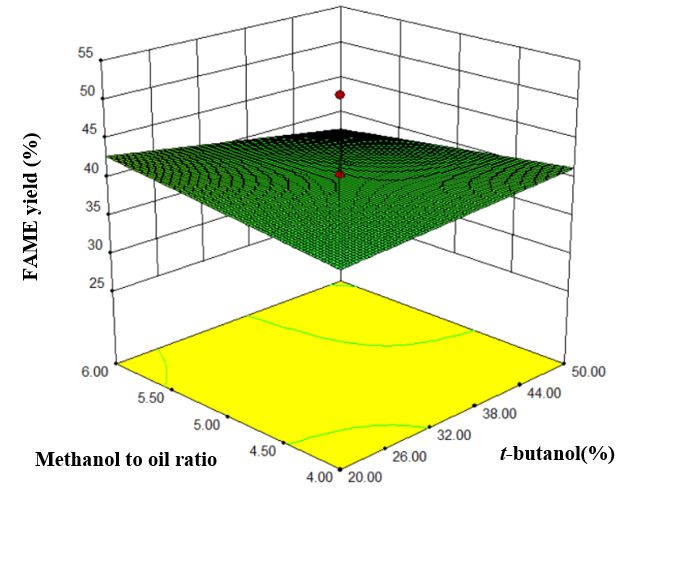


Fig.5S. Response surface curves showing the interactions for CALB
